# Enabling Technologies for Urban Smart Mobility: Recent Trends, Opportunities and Challenges

**DOI:** 10.3390/s21062143

**Published:** 2021-03-18

**Authors:** Sara Paiva, Mohd Abdul Ahad, Gautami Tripathi, Noushaba Feroz, Gabriella Casalino

**Affiliations:** 1 Instituto Politécnico de Viana do Castelo, 4900-367 Viana do Castelo, Portugal; sara.paiva@estg.ipvc.pt; 2 Department of Computer Science and Engineering, Jamia Hamdard, New Delhi 110062, India; gautami1489@gmail.com (G.T.); noushaba.feroz@gmail.com (N.F.); 3 Department of Computer Science, University of Bari Aldo Moro, 70125 Bari, Italy

**Keywords:** smart mobility, sustainability, smart cities, smart services

## Abstract

The increasing population across the globe makes it essential to link smart and sustainable city planning with the logistics of transporting people and goods, which will significantly contribute to how societies will face mobility in the coming years. The concept of smart mobility emerged with the popularity of smart cities and is aligned with the sustainable development goals defined by the United Nations. A reduction in traffic congestion and new route optimizations with reduced ecological footprint are some of the essential factors of smart mobility; however, other aspects must also be taken into account, such as the promotion of active mobility and inclusive mobility, encouraging the use of other types of environmentally friendly fuels and engagement with citizens. The Internet of Things (IoT), Artificial Intelligence (AI), Blockchain and Big Data technology will serve as the main entry points and fundamental pillars to promote the rise of new innovative solutions that will change the current paradigm for cities and their citizens. Mobility-as-a-service, traffic flow optimization, the optimization of logistics and autonomous vehicles are some of the services and applications that will encompass several changes in the coming years with the transition of existing cities into smart cities. This paper provides an extensive review of the current trends and solutions presented in the scope of smart mobility and enabling technologies that support it. An overview of how smart mobility fits into smart cities is provided by characterizing its main attributes and the key benefits of using smart mobility in a smart city ecosystem. Further, this paper highlights other various opportunities and challenges related to smart mobility. Lastly, the major services and applications that are expected to arise in the coming years within smart mobility are explored with the prospective future trends and scope.

## 1. Introduction

Smart mobility is an emerging concept that is increasingly aligned with sustainable world development, taking into account the 17 sustainable development goals set by the United Nations [1] for 2030. The concept of mobility, and how it will articulate with the planning of cities and the logistics of transporting goods and people, will experience drastic changes in the coming years. Demographic growth continues to follow an exponential route, with 6 billion people registered in 1999, an approximate number of 7.7 billion in 2020 and a predicted number of approximately 9 billion expected for 2040 [2]. This growth we see in cities all over the world will necessarily translate into the need for new route optimization algorithms for vehicles and people, traffic management to reduce congestion, and greater optimization in logistic processes, among others. However, the concept of smart mobility goes far beyond solving these problems since future contributions are expected to represent differentiating and truly innovative solutions [3]. The focus on the sustainability of the solutions developed, active transport, the use of environmentally friendly fuels and engagement with citizens are aspects that should be part of smart mobility in the coming years. The impacted dimensions will therefore be varied and include sustainability, economy and living, which has a direct impact on citizens and also on government entities [3].

Two key concepts are at the base of what will be the evolution of smart mobility in the coming years: on one hand, the transport of goods, and on the other, the paradigm shift in the mobility of people that will transition to mobility-as-a-service. Regarding transports of goods, we already witness several robot prototypes around the world that deliver basic necessities to people’s homes, which will mean an adjustment to how businesses operate (supermarkets, delivery services, among others). Internet of Things (IoT), Big Data and Artificial Intelligence (AI) will play a fundamental role in new solutions. It should be highlighted that the changes the coming years will bring will promote inevitable changes in what will be the jobs of the future. Regarding the transport of people, trends are evolving towards a mobility-as-a-service paradigm, with a drastic reduction in the number of private vehicles that will be filled by electric, shared, lighter and smaller vehicles and with autonomous driving.

This paper provides an exhaustive literature review of several solutions across multiple domains that are related to smart mobility in smart cities. The paper also provides an overview about what is smart mobility and the related opportunities and challenges. Further, the paper highlights the enabling technologies that are being used in order to deliver smart services to citizens. Lastly, the future trends and conclusions are presented.

### 1.1. Need and Importance of Smart Mobility

**Figure 1:**
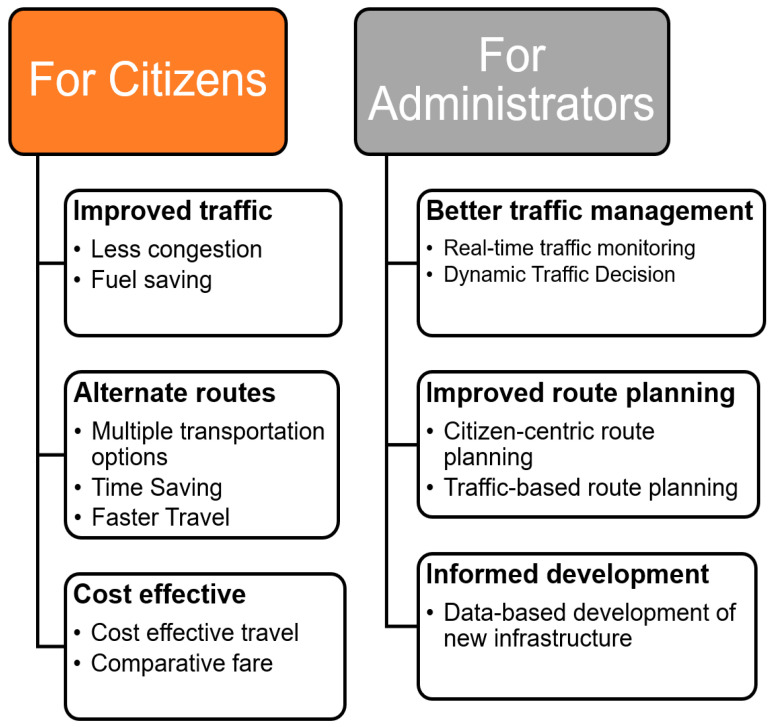
Need and importance of smart mobility.

Smart mobility enables inhabitants to navigate and move freely within the smart city surroundings. Improved traffic management, the availability of alternative routes (in case of traffic or emergencies), and dedicated routes and navigation for essential services (such as ambulances, government vehicles, official movements) can be facilitated by smart mobility. Such mobility services are needed to provide congestion free, environment friendly and sustainable alternatives for the inhabitants and administrations alike. With smart mobility solutions we can have two-fold advantages both for citizens as well as administrations, as shown in Figure 1.

### 1.2. Paper Organization

**Figure 2:**
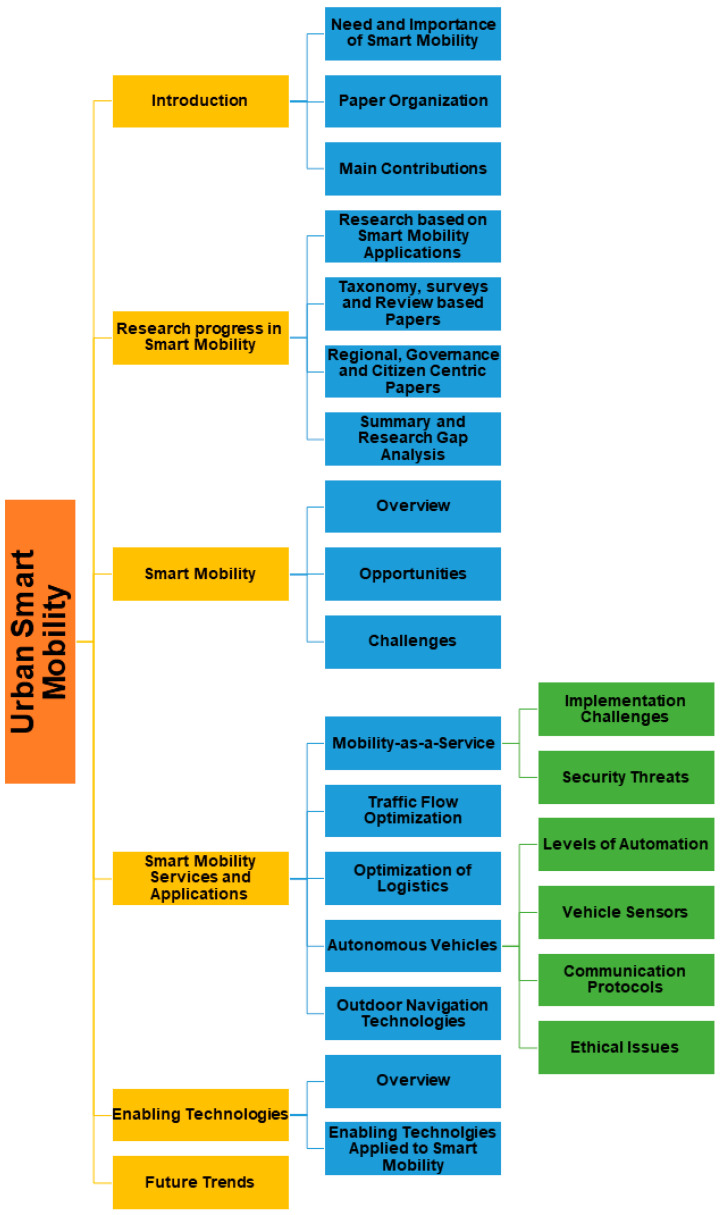
Overview on the paper organization.

This paper is organized into seven sections. The second section presents recent developments within the smart mobility domains and also identifies the research gaps and open issues. The third section presents a description of smart mobility and its role in smart cities. After an overview, we present the main opportunities and challenges for the adoption of smart mobility in the coming years. The fourth section presents some applications and services of smart mobility such as Mobility-as-a-Service, Traffic Flow Optimization, Optimization of Logistics, Autonomous Vehicles and Outdoor Navigation Technologies. The fifth section presents enabling technologies for smart mobility and their role in realizing smart mobility solutions, namely the contribution of Internet of Things, Big Data and Artificial Intelligence. Finally, we present future trends and main conclusions in the sixth and seventh sections, respectively. An overview of the paper organization is shown in Figure 2.

### 1.3. Main Contributions

The major contributions of this paper include:

- A comprehensive review of smart mobility solutions and related services proposed in the last few years.
- The contextualization of how smart mobility is framed within smart cities and its key benefits and attributes.
- Discussion on the opportunities for smart mobility to become a reality in the coming years as well as the major issues and challenges it its realization.
- Meaningful insights into the future of the mobility-as-a-service paradigm.
- Overview of the Enabling Technologies that will support smart mobility services and applications such as AI, IoT, blockchain, geospatial technology and big data.
- Future trends for smart mobility.

## 2. Research Progress in Smart Mobility and Gap Analysis

**Figure 3:**
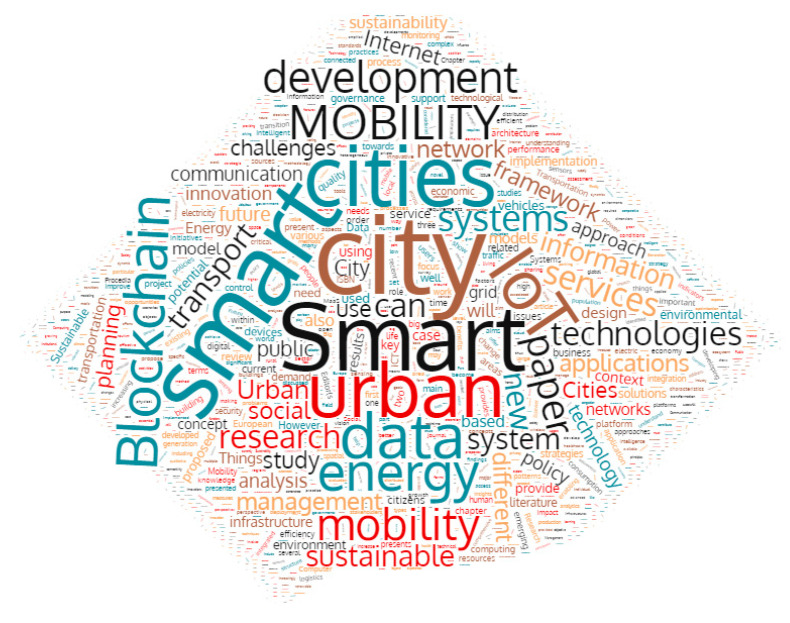
Word cloud of search query results.

This section provides a detailed description about the research progress made in the field of smart mobility. We also try to identify the research gaps in the existing literature. In order to conduct the survey, we have adopted the standard systematic literature review methodology [4]. Firstly, we performed a keyword based search using the online research paper databases Science Direct (https://www.sciencedirect.com/ accessed on 15 January 2021) and IEEE Xplore (https://ieeexplore.ieee.org/Xplore/home.jsp (accessed on 15 January 2021)). We primarily used the keyword “Urban Smart Mobility” to search for articles. We received 851 results from IEEE Xplore and 10,439 results from Science Direct. We refined the search criteria by adding the terms “Smart Cities” along with smart mobility, which reduced the results to 440 (IEEE Xplore) and 8217 (Science Direct). We further refined the criteria by adding “Enabling technology” along with the previous two keywords, which further reduced the results to a total of 2486 papers. Next, refinement was performed on the basis of publication year. We analyzed the papers from 2011 to 2020. This refinement reduced the number of search results to 2049. We then filtered these articles based on the title contents to eliminate articles that were out of the scope of this review. Finally, 294 articles were left and a further selection has been made based on the abstract contents. Indeed, we read the abstracts of these 294 articles to further filter out articles which are not related to our prime focus. After this step, 186 articles were left. These 186 articles were read thoroughly to understand their key proposals, discussions and arguments. Finally, only 81 articles, which completely matched our criteria, were considered for the analysis. These articles were further analyzed and discussed to identify their key findings and research gaps. Figure 3 shows the word cloud of the results obtained by the search query terms.

**Figure 4:**
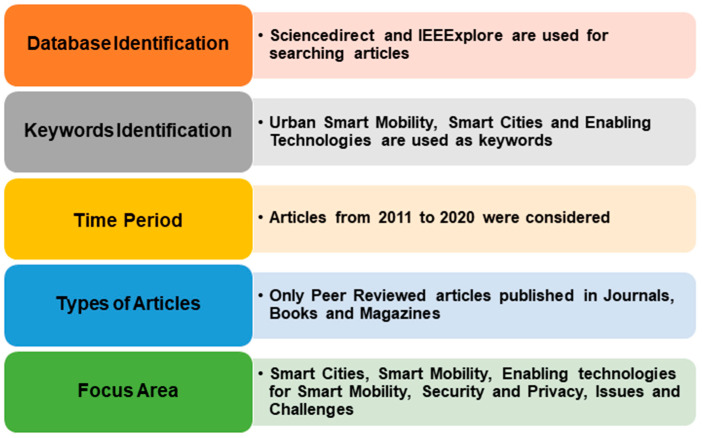
Steps in methodology adopted.

Figure 4 shows the steps involved in the review methodology that has been used:

1. Classification of Articles on the basis of the type: we have chosen only peer reviewed research, review and survey articles satisfying our criteria;
2. Identification of Publication Year: articles from 2011 to 2020 only were analyzed in the literature review;
3. Classification of Articles on the basis of keywords: we have combined the following keywords
  - Smart City
  - Urban Smart Mobility
  - Enabling Technology.

Once the articles were filtered, a thorough study was conducted to identify the crux and key findings of each paper. The details about the state-of-the-art are discussed below.

### 2.1. Research Based on Smart Mobility Applications

This section specifically discusses the articles on the applications of smart mobility for the citizens of the smart city. These papers include framework based, application and deployment based systems.

In [5], the authors have presented a smart mobility application “UTravel”, which is based on “Universal Profiling and Recommendation (UPR)”. The UTravel application is based on context awareness and user profiling and recommends the optimal points of interest (POIs) depending on the user’s location. The application has been developed and deployed on both Android and iPhone platforms. To evaluate the performance of the application, the authors have conducted real-world as well as simulated experimentations and observed that the recommendations provided by the proposed system exhibit high precision, coverage and recall. A traffic control system based on cooperative agents is presented in [6], which aims at reducing traffic jams at road intersections. For modelling road intersections, the authors have used smart agents, viz. “View agents” (to count cars), “Traffic Light agents” (to control the duration of traffic lights) and “Intersection agents” (to control the duration of a particular traffic light). The system has been developed for settings where several traffic lights operate in collaboration, i.e., “Infrastructure-to-Infrastructure (I2I)” communication. The system has been implemented using “Java Agent Development Framework (Jade)” and validated using “Simulation of Urban MObility (SUMO)”. The results show that the proposed system performs better traffic congestion reduction than “Smart Traffic Lights System (STLS)”. In [7], the authors intend to evaluate and identify smart mobility actions taking into account their Information and Communication Technology (ICT) content and objectives. The authors have introduced a novel action taxonomy involving a systematic approach to smart mobility and analyzed the role of ICT in improving the quality of life of people, increasing the public value and promoting smart mobility actions. A survey has been conducted that identifies three stages of smart mobility actions, viz. “Starting”, “Intermediate” and “Mature”. Moreover, the authors state that smart citizens are crucial for implementing a viable, successful and sustainable smart mobility framework. A framework for developing and implementing smart mobility applications, “Smart Mobility for All (SMAll)”, is given in [8], which leverages the concept of “microservices orchestration” to improve the handling of multiple data sources and the creation of novel services. The proposed system is aimed at assisting elderly and/or disabled persons to move in an urban setting. To do this, a network of taxis is deployed to fulfil the need of each applicant and SMAll synchronizes the involved taxi operators, sometimes combining calls that are nearby in an effective manner. The authors maintain that the deployment of SMAll results in improved quality of service with lower administrative expense. To handle the issues regarding the security, privacy, scalability and management of “Smart Mobility Data-market (BSMD)”, a six-layer Blockchain architecture is proposed in [9]. The architecture enables the exchange of encrypted data to the blockchain among users complying with the rules of the transaction provided by the data owner. The authors have assessed the performance of BSMD over a 370-node blockchain and established that BSMD safeguards the users’ privacy and security by offering data access management control and preventing message interception and spoofing. Within the bounds of mobility, the authors have classified three important elements in a general blockchain, viz. “shared ledger”, “peer-to-peer network” and “consensus mechanisms”. Smart contracts are added into BSMD nodes to control access to the shared mobility information. In [10], the authors have presented a framework to aid in the efficient designing of a traffic light network in an urban setting to minimize traffic jams. The framework “HITUL” assists in the decision making of traffic control management by determining the ideal traffic light schemes by employing micro-simulations and bio-inspired methods. The authors have evaluated HITUL through a case study of the Spanish city of Malaga and observed that it is an effective technique towards minimizing traffic jams. Moreover, the authors have used “Simulator of Urban Mobility (SUMO)” to obtain arrangements of practical scenarios based on actual mobility trends in a city. In [11], the authors attempt to demonstrate the spectrum of development of “Spanish Smart City” measures with a view to mobility and environmental concerns. The authors have conducted a study in 62 cities of “Spanish Smart Cities Network (RECI)” and provided a synergistic map displaying a comprehensive evaluation and advancement of the cities on the basis of demographic and socioeconomic variables. The findings indicate that smart mobility is a vital element of smart cities and smart environment has poor outcomes in Spanish cities. A novel approach to predict the occupancy rate of car parking space has been given [12], which is premised on deep learning with Recurrent Neural Networks (RNNs). The authors have presented two metaheuristic techniques to optimize the performance of the RNN design, one based on Genetic Algorithms (GA) and the other based on Evolution Strategy (ES). The authors have examined the occupancy rates of 29 car parks in Birmingham, UK and observed that the proposed approach is more useful and exceeds the performance of available competition.

### 2.2. Taxonomy, Surveys and Review Based Papers

A taxonomy for the formulation of smart city services is presented in [13]. For this purpose, the authors have analyzed the latest relevant works covering 42 services provided by 9 smart cities globally. The proposed taxonomy is eight-dimensional and offers a standard vocabulary to facilitate communication regarding the services. The taxonomy is aimed at aiding policymakers and researchers in the further improvement of the area. The authors have incorporated general definitions, concepts and illustrations for each dimension and specified service. In [14], the author has presented a novel action taxonomy concerning an extensive methodology related to smart mobility. A thorough survey has been performed covering 114 works regarding the influence of smart actions on the quality of life of citizens, the expectations of stakeholders, regularly applied smart mobility measures and the role of ICT in smart mobility. The survey has resulted in the identification of three smart mobility action phases and six smart mobility goals. Moreover, the paper notes that though ICT is not essential for the implementation of smart city actions, it is important when smart mobility actions become increasingly complex, integrated and extended. The authors have proposed a distributed adaptation method for ensemble-based systems in a smart mobility context in [15]. They have presented the “Collective Adaptation Engine (CAE)” that can address multiple issues collectively in a feasible and scalable manner. To assess the proposed model, the “DeMOCAS” framework has been used to simulate the context of urban mobility. The findings indicate that CAE solves urban mobility challenges and promotes sustainable mobility. In [16], the authors have proposed the application of system dynamics to simulate substitutes for conventional human mobility. An analysis of six scenarios is presented for attaining optimized decision-making within organizations, defining the profiles of travelers and achieving sustainable smart mobility with the eventual goal of enhancing the citizens’ quality of life. The analysis is aimed at understanding the principal dynamic interactions between all the variables of the system and managing their complexity. A detailed and realistic structure has been introduced in [17] to design a comparative analysis, which gauges cities based on the smartness of their transport frameworks. The authors have gathered data from 26 cities globally, identified 66 indicators of smartness and observed that cities such as Seattle, London and Sydney have the most advanced smart transportation, with London having the best emergency transport facilities, Singapore and London having the best public transport facilities, and Paris and Seattle having the best private transport facilities. In [18], the authors have analyzed the likely transition of present issues of mobility governance with the aim to safeguard and increase the public value. Four case studies have been conducted to study distinct mobility governance issues and an analytical structure has been presented to evaluate the smart mobility governance novelties. Furthermore, the authors assert that the transition in mobility governance should be backed with technological transition. In [19], the authors have focused on enhancing a vital element of smart mobility, viz. positioning in a smart university setting acting as a representative for a smart city. An indoor positioning system integrated with an outdoor positioning system has been proposed and implemented to facilitate persistent indoor and outdoor navigation. The authors have implemented the proposed framework on the smart university platform, “SmartUJI”, at Universitat Jaume I, Spain. Moreover, two mobile applications have been created and implemented by the authors—“SmartUJI APP” (to provide map-based information regarding various campus services) and “SmartUJI AR” (to provide communication with the campus via an augmented reality interface). The evaluation of the two applications indicates their useful assistance to visitors, faculty and students in enhancing spatial position and finding university facilities. The authors have specified a range of mobility indicators to assess smart urban mobility in [20]. They have presented a relevant quantitative methodology that is applicable to any city globally with the aim to benchmark smart cities. The authors have selected Italian cities for the evaluation of sustainable transportation to gather crucial data, and they observed that the city of Turin is the best with regard to smart transport. Moreover, it is noted that the northern cities of Italy have overall better ranking than the southern cities. The author has focused on the transformation from an automobile community to a multimodal community in [21], fostered by the advent of smart mobility powered by ICT. The paper highlights three outcomes obtained from quantitative analysis of data from the German region of Rhine-Main. These include transport poverty, multimodal divide and critical thinking as factors contributing to mistrust towards multimodal mobility, and the paper recommends a change in this perspective to enhance the debate surrounding multimodality. Eleven metropolitan cities in Italy have been studied by the authors in [22] to assess the possibility and degree of applying the smart city model with the aim to boost the effectiveness and living conditions of urban areas. The authors have identified key smart city measures and parameters and grouped them to underline the impact of the smart city model on the mobility systems and observed that a poor starting place limits its implementation. The paper classifies the smart mobility paradigm into three groups, “accessibility”, “sustainability” and “ICT”, and applies 28 parameters to locate contexts with the best accessibility and sustainability. Furthermore, it is indicated that ICT is ineffective when the transport framework is inadequate. A distributed system for mobility data management and semantic improvement has been proposed in [23], which would benefit the areas of traffic management, m-health, urban kinetics examination and emergency management, among others. The proposed system, “SemanticMOVE”, enables the semantic management of mobile things, and offers understanding of the mobility semantics as well as the identification of potential user movements, behaviors and activities. In [24], the authors have focused on the importance of a multi-disciplinary and collective methodology to smart mobility, which would enable the shift to a “smarter mobility” to improve city development and citizens’ quality of life. A study has been conducted in Belgium to analyze the advancement of smart mobility from techno-centered to user-centered. The authors argue that although solutions to smart mobility challenges are sought in novel technologies, these solutions are not absolute and smart mobility outgrows technology and users. In [25], the author has highlighted the relationship between smart mobility and social sustainability and clarified the definitions of the two concepts. The paper indicates that the social sustainability of smart mobility depends on the path taken by the latter. Two distinct scenarios have been given, the first of which involves increased ease of use promoting the prevalence of cars, resulting in social discord, inequity, the scarcity of car driving and parking spaces, etc. The second scenario involves the application of technologies to enable services such as ride sharing and on demand rides, resulting in negative consequences on transportation workers but a positive impact on social harmony, equity and availability. A theoretical assessment of experimental governance is presented in [26] through the analysis of fundamental literal and practical assumptions with respect to smart mobility. The authors have selected an experimental study of smart mobility in Sweden to examine experimental governance as a policy tool to facilitate the objectives of public stakeholders and assert that these stakeholders have a special role in experimental governance. In [27], the authors have presented a quantitative approach for assessing the urban mobility in the Italian area of Cagliari and recommended the roadmap to achieve the finest mobility globally. The advantages of smart mobility have been analyzed in Cagliari and similar urban areas by choosing indicators based on the relevant data of the selected contexts. The data for the indicators have been collected from the year 2014 and classified into groups, and it is observed that mobility data sharing is not adequate so far. In [28], the author has explored the framework of citizen engagement and efficiency of Japanese Smart Communities to contemplate the collaborative design and development of a smart mobility framework. The paper indicates the anticipation of little feedback from the citizens and the deployment of ICT to direct the participants and modify their behavior. It is observed that the current smart city initiatives are in a nascent phase to comprehend that the objectives of the administration, focused on energy saving or developing alternate energy sources, have limited ICT deployment and the ICT related projects are poorly coordinated. A holistic methodology to model the efficiency of public transport facilities is given in [29], schemed as a whole in a multi-stakeholder context from an end-to-end perspective. The authors seek to underline the key aspects of the quality of service from distinct viewpoints and have provided a holistic methodology to modelling that supports the quality of service design, implementation and monitoring in smart transport. Moreover, the paper presents “UCoMS” for the analysis of the quality of service, and the proposed methodology has been validated in the Italian region of Apulia. In [30], the author seeks to analyze the meaning of “smart” in the context of smart urban mobility and the relationship between smartness and sustainability. The author has uncovered discord and insufficiency in the literature pertaining to smart urban mobility and has presented and analyzed the definition of smart urban mobility. The paper attempts to bridge the gap between the concepts of smart and sustainable for the growth of urban mobility. A comprehensive analysis of the role played by “Intelligent Transport Systems (ITS)” in assisting urban smart mobility is given in [31]. A total of 71 papers have been analyzed, ranging from 2006 to 2014, with 34% case studies and 21% simulations. The case study-based works analyze the deployment of ITS in urban cities, while the simulation-based works analyze the influence of ITS on urban mobility and measure cost, time and environmental impacts. The paper aims to detect the gaps in the literature and observes a generic insufficiency of quantitative frameworks. The authors recommend a roadmap for future research based on the identified inadequacies.

In [32], the author highlights the factors that link citizens to different facilities, especially mobility and ICT frameworks in the Senegalese city of Dakar. It is observed that motorized methods amount to 40% while non-motorized methods amount to 60% of the overall mobility, and the public mobility sector is largely informal. The author has noted the measures taken by the administration to build a positive setting for ICT development and deployment, including E-Infrastructure, E-Education and E-Governance.

### 2.3. Regional, Governance and Citizen Centric Papers

A case study in the Portuguese city of Lisbon has been presented in [33], which establishes a performance assessment of passenger and commercial vehicle redirection. The results indicate that redirecting vehicles not only reduces travel duration, but increases the road effectiveness in the urban grids and the traffic output in the analysis of routes. Moreover, it is observed that the effectiveness is higher at the route level (variation of 16–32% in travel and 4–13% in delay time) than at the network level (average variation of 2% in travel and 6% in delay time for 10% driver conformity rate). The concept of “Vehicular Social Networks (VSNs)” has been given in [34], focusing on the importance of highly efficient and secure smart city transmission in VSNs. The paper proposes a use case on trajectory data-analysis-based traffic anomaly detection for VSNs and highlights VSN-related research challenges and potential solutions to facilitate the realization and extensive application of VSNs. In [35], the authors aim to establish an indicator for assessing the degree of smart mobility solutions deployed in urban areas. The paper indicates that the insufficiency of comprehensive knowledge related to the evaluation of certain ratings of the existing literature and the inordinately general analysis of smart mobility challenges have prompted the need for the indicator. The proposed indicator is intended for facilitating the analysis of mobility scenarios in accordance with the notion of smart cities, enabling comparisons in varied urban areas for the identification of best practices to promote the advancement of smart mobility. An analysis of “Mobility as a Service (MaaS)” has been presented in [36] to evaluate its potential impact for city policymakers with regards to governance and sustainability. The paper stresses that MaaS is not a fixed commodity but a conceptual way of providing services to customers. Moreover, the potential risk to mobility and social sustainability due to over dependence on single operators of novel services and the potential impact of novel services on current services are given. In [37], the authors have focused on evaluating the smartness of transport systems in the cities of Ghana and illustrating the realization of the notion. It is observed that Ghanaian cities are predominantly reliant on roads for the mobility of people and cargo, and the swift increase in the number of vehicles accompanied by insufficient expansion of road networks has weakened the efficiency of urban areas. The paper implies that the lack of smart mobility in these cities endangers their sustainability and, as a result, necessitates investment in mobility systems together with increased reception and technological awareness among the citizens. A general framework for the implementation of fog computing features in a “Vehicular ad-hoc Networks (VANET)” context is introduced in [38]. The proposed architecture has been applied in two Fog applications, one for the identification of traffic aberrations, and the second for predicting bus arrival time to supply passenger information. The evaluation results indicate that the outcomes of these two applications are akin to those offered by Cloud, the information offered is faster, reliable and real-time, and the overall traffic is reduced. In [39], the authors have discussed the “autonomic transport management system”, which is an ICT based system for the management of transport. The presented approach enables the formation of a “P2P-Overlay Network’ on the existing networks and applies IPv6 along with multicast and transparent routing for the effortless expansion of the network to the masses. The methodology enables users to join the network using their personal devices as well as the integration of infrastructures such as traffic management, traffic lights, trains, etc., into the overlay network with ease.

An analysis of existing IoT methods and notions concerning smart cities and smart mobility is presented in [40]. Moreover, an analysis of different properties and uses related to smart mobility and real-time traffic management systems has been given. The paper identifies and addresses the major challenges related to smart cities and smart mobility, such as unequal geographic advancement, privacy concerns and the lack of collaboration. Furthermore, significant gaps have been identified in the domain of smart mobility regarding “Vehicular Ad hoc Networks (VANETs)” and “Smart Traffic Lights”. In [41], the authors have illustrated the implementation of “Service-Dominant Business Model Radar (SDBM/R)” in the context of smart mobility. The proposed framework is aimed at designing mobility related business models for the mobility of travelers and cargo in a collaborative fashion. In a multi-stakeholder business context, the authors have designed the system as a central element in the development of complex digital novelties that provide value to users. A survey has been conducted to validate the model and the input from the participants indicates the relevance of the proposed model and its potential for practical use. In [42], the authors have explored the correlation between urban intelligence and sustainable mobility forms for the municipalities in Australia, to explore whether the intelligence of cities contributes to sustainable mobility forms. The impact of growing broadband internet availability on sustainable mobility mode is analyzed using a multivariate multiple regression model, and it is observed that growing broadband internet availability decreases the use of active, public mobility means, while increasing the use of private commuting means. An analysis of the association between the deployment of the smart city notion and the sustainable mobility notion is presented in [43]. The authors have also analyzed the effect of carbon dioxide release from smart city components as a determining factor of mobility. The United Nations’ “ForFITS (For Future Inland Transport Systems)” model has been used to predict the possible carbon dioxide release resulting from the implementation of the Warsaw transport system. The findings indicate that a thorough change in the mobility and energy domains is necessary to achieve the reduction goals identified by the “European Union 2011 White Paper on Transport”. In [44], the author has focused on the notion of shared mobility and has presented an analysis of the present literature. The paper observes that most of the literature is aimed at evaluating the effect of shared mobility on factors such as modal transition, congestion, transit clientele, vehicle possession and environmental aspects, while little consideration is given to female safety, accessibility and comfort during travelling, revealing a growing gender parity in urban mobility.

### 2.4. Summary and Research Gap Analysis

This section summarizes an overview of the state-of-the-art about smart urban mobility and identifies the research gaps and open issues within its domain. For ease of comprehension, Table 1 summarizes the works covered in the literature review.

Authors in [5,6] primarily focused on describing the benefits of smart cities for the users and how smart mobility can be used as an aid to solve traffic congestion, urban planning, recommendation systems, etc. In [7], it was concluded that responsible behavior and the attitude of the inhabitants have a positive effect on smart mobility initiatives. The security aspects of smart mobility were discussed in [8,9]. Some of the key points which were found missing in the reviewed literature include:

- User Privacy
- Data Integration issues
- Data Standardization issues
- Sensor characteristics
- Impact of external environment on sensing capabilities of sensors.

In this paper, we have provided a holistic review which covers the above aspects in addition to those previously discussed. User privacy concerns and a lack of technical and operational knowhow restrict common people, especially elderly people, women and laborer workers, from using smart mobility services [45]. Several privacy preserving approaches have been developed in recent years for the effective adoption and ease of use of mobility services [46,47]. The governance issues in smart mobility services, including consent-based data capturing, manipulations and usage, data integration and standardization challenges, were covered in [48,49,50,51]. The importance of sensor characteristics for sensing and capturing the data about the subject and its surroundings plays a vital role in developing effective and intelligent mobility initiatives [52,53,54,55]. The impact of external environments on the performance of the sensors is an essential field of study, as discussed in [56,57]. The materials used in the development of sensors also play an important role in deciding their usage and life [57].

Apart from these, a few open issues which are still prevalent in smart mobility include:

- Enforcing uniform and ubiquitous mobility laws, rules and regulations
- Citizen participation in mobility initiatives
- Crowdsensing in smart mobility
- Interoperability
- Legacy Infrastructure setups
- Amicable Cooperation between public-private mobility services players.

With the rapid technological transformations, these open issues will surely be addressed in years to come. As the smart city initiative is slowly becoming a reality, extensive research is being conducted to handle such open issues [58,59].

## 3. Smart Mobility in Smart Cities

### 3.1. Overview

Urban mobility is one of the major components of a smart city that acts as a critical factor behind smart and sustainable development. Today, nations across the globe are heavily investing in transport infrastructure as an approach to ensure smart and affordable means of transportation to citizens. Smart mobility is one of the key defining features of the smart city [60]. In most developing nations, where rapid urbanization is increasing the demands of smart and cleaner modes of transport, the need for smart mobility is ever increasing. The key mobility challenge in such countries demands the mass adoption of public transport systems. The key attributes of the smart mobility concept, as shown in Table 2, allow the users and other stakeholders clean, safe and efficient travel.

**Figure 5:**
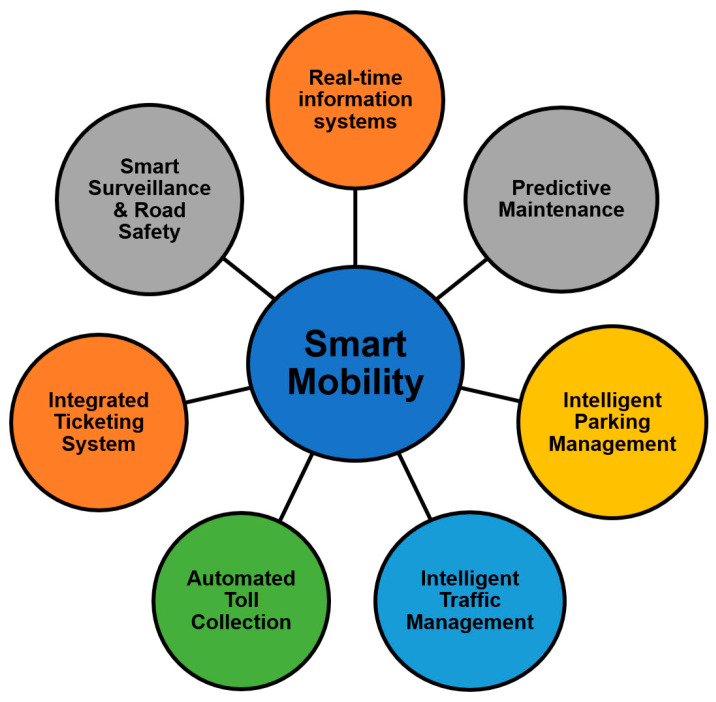
Key benefits of using smart mobility in a smart city.

The concept of smart mobility encompasses the shift from the traditional transportation system to Mobility as a Service (MaaS), where intelligent infrastructure connects various stakeholders and entities to provide an efficient, intelligent and sustainable solution [61]. It includes multiple modes of transportation including on demand mobility solutions, electric vehicles, bikes, rapid mass transit facilities, walking, etc. The key idea behind smart mobility is to ensure quality service to the citizens and at the same time minimizing the impact on the surrounding environment [62]. Figure 5 presents some of the key benefits of integrating smart mobility solutions in a smart city ecosystem.

Real Time Information Systems: The use of emerging technologies in the existing transportation systems and the development of smart mobility solutions provide real time data collection, monitoring, and management. The data collected from various connected entities result in efficient and smart information systems.

Predictive Maintenance: Further, the machine learning and artificial intelligence concepts can be utilized for the predictive maintenance of various processes in advance. This is facilitated by continuous data collection and monitoring.

Intelligent Parking Management: The real time data collected from sensors and other connected devices can be analyzed to provide useful insights into the availability of parking slots at various locations.

Intelligent Traffic Management: The use of efficient data analytics can help in the real time monitoring and management of traffic to avoid any jams and congestions. Further, real time notifications can be sent to the connected vehicles regarding the state of parking, routes, etc.

Automated Toll Collection: The smart mobility solutions provide hassle free movements at toll plazas by facilitating automatic payments.

Integrated Ticketing Systems: The Mobility as a Service concept provides the integration of several local services, thus facilitating a smart ticketing system to provide easy and multimodal services to citizens.

Smart Surveillance and Road Safety: The cameras and other security devices forming a part of the connected network help in monitoring the state of traffic, thus enhancing road safety.

**Figure 6:**
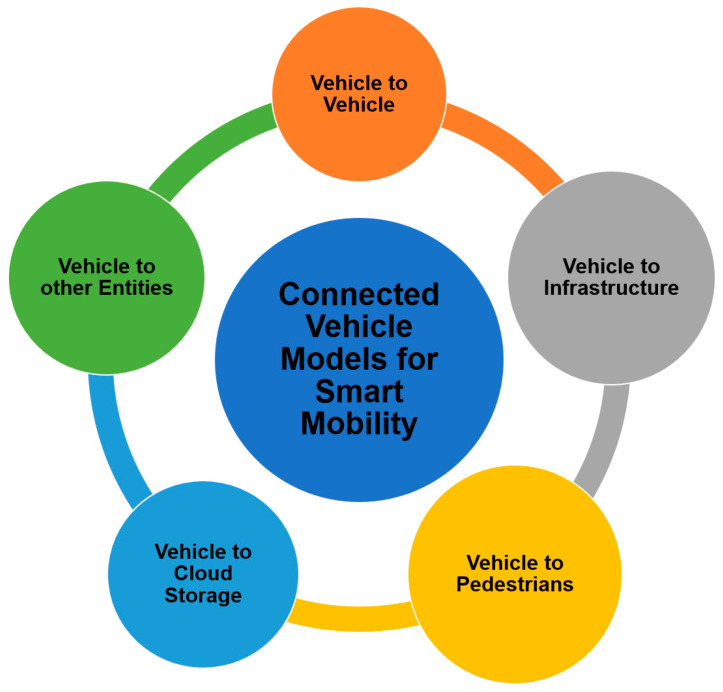
The primary connected vehicle models for smart mobility.

The emergence of connected technologies has opened new possibilities for urban planning and management. The economical and human cost associated with road mishaps is profound. Today, intelligent transport management systems have enabled the concept of Smart Mobility, making driving and commuting safer and more efficient. The connectivity models for smart mobility include the communication between the vehicles and the other connected entities, as shown in Figure 6.

Vehicle to Infrastructure: Vehicles are connected to the traffic infrastructure such as traffic lights, tolls, parking, pedestrian crossings, etc., to enable the sharing of real time traffic information and predicting traffic jams and delays.

Vehicle to Vehicle: Vehicles are connected to other vehicles to ensure road safety. The data shared between vehicles further help in avoiding congestion and parking management.

Vehicle to Cloud Storage: Vehicle to storage connectivity helps in real time information sharing, storage and processing.

Vehicle to Pedestrians: This concept connectivity model connects vehicles to pedestrians via smart devices such as mobile phones and wearables to facilitate pedestrian safety and offer real time efficient mobility options and solutions.

Vehicles to Other Entities: This ensures a comprehensive connectivity model that integrates smart mobility with other smart city components and processes.

### 3.2. Opportunities

Smart mobility solutions can bring unprecedented benefits for the smart city ecosystem. Smart traffic management to intelligent land use planning and management can be made possible with the help of smart mobility solutions. City planners can exploit the intelligent mobility approaches to come up with effective and efficient design plans which can be realized in sustainable and environmentally friendly ways. There are several opportunities associated with smart mobility approaches for the city planners, authorities, users and other inhabitants of the smart city [5,6,7,8,9,10,11]:

Strategic Route Planning and Development: The Big Data generated from the ubiquitously connected IoT devices can be harnessed to extract better insights about the traffic and city routes. This information can further be used for strategic route planning and development.

Business Marketing Campaigns—Intelligent Ads Placement: Businesses can use digital sign boards and hoardings to effectively place their marketing advertisements at critical locations (where they can be made visible to maximum vehicles/traffic).

Digital Ads and Revenue Generation: The rudimentary poster and banner-based advertisements can be replaced with digital counterparts. This has two-fold benefits. One, it reduces paper wastage, and secondly, since it can be dynamically updated with simple coding/programming, it consumes less time for setup and updates.

Alternate Route Management and Incentivizing Citizens for Cooperating in Decision Making and Following Instructions: law makers can take real-time data-based dynamic decisions for traffic management. This information can be shared with commuters to avoid possible traffic congestions by suggesting alternative routes. Governments can also incentivize the law-abiding citizens who help in following and enforcing these laws. This step can promote citizen centric participative governance.

Opportunities for Builders, Businesses and Manufacturers: Builders, businesses and manufacturers can come up with innovative solutions for providing sustainable and environment friendly alternatives for the classical approaches. The area/techniques focused startups can exploit this opportunity for growing their businesses.

Cheaper and Multiple Options for Transportation: With smart mobility solutions in place, citizens have the option for a better quality of service (QoS) in terms of comparative costs, improved and multiple options for transportation and hassle-free commuting experience.

Improved Serviceability: Data analytics approaches can be applied to provide citizen centric services to the inhabitants of the smart city. Since the decisions and policies are data driven, they can surely improve the QoS.

### 3.3. Challenges

Some of the main challenges that are presented to smart mobility nowadays include:

Infrastructure: Implementing smart mobility solutions in a smart city system has high infrastructural demands to overcome the pressure on the suboptimal transportation systems in most parts of the world. The increasing popularity of self-driving and electric vehicles requires network connectivity, high bandwidth and electric charging stations. To fully utilize the potential of smart mobility, there is a need to develop the infrastructure that can realize the concepts surrounding smart systems.

Last Mile Connectivity: One of the major issues in public transportation systems is the low-cost last mile connectivity. For efficient smart mobility solutions, there is a need for door-to-door connectivity irrespective of the mode of transportation.

Security and Privacy: The need for connected devices and the rapid generation of rich personal data pose serious privacy concerns related to data sharing amongst devices and users. Moreover, due to the connected devices, the entire network is vulnerable to outside attack and breaches.

Governance: The concept of smart mobility has extended the scope of conventional transportation systems to include other stakeholders such as tech companies and service providers. The inclusion of these new actors requires modified policies and rules governing the smart mobility. The regulations governing smart mobility systems are still lagging behind. With no standards governing the use of smart mobility solutions, the mass adoption of these smart solutions is still far from reality. There is a need to seamlessly integrate the existing traffic laws to meet the demands of smart mobility solutions.

Initial Adoption: One of the major issues with smart transportation is creating awareness amongst the potential users for its adoption. The majority of solutions are in the early stages of development, which does not provide significant proof of the exact goals of the mobility solutions.

Dynamic Routing and Transportation Mobility: The efficient solutions require dynamic routing systems to estimate the travel demands of the users and optimize the available resources to provide the solutions. This process requires sophisticated software and technological solutions.

Network Management and Monitoring: The smart ecosystems comprise millions of connected entities, which makes the network management and monitoring complex and costly.

Data Acquisition and Integration: The data are collected from heterogeneous sources with different security and privacy protocols. Handling the large volumes of real time data generated from the connected devices is a complex task.

Legal Challenges: The involvement of multiple stakeholders such as payment companies, governments, city administrations, public-private transportation, users, etc., requires a well-defined legal system and support policies for smart mobility.

## 4. Smart Mobility Services and Applications

### 4.1. Mobility-as-a-Service

The emergence of the Mobility-as-a-Service (MaaS) paradigm is a trend that is expected in the coming years in the area of mobility in smart cities. The current reality of door-to-door journeys seems to have its days numbered as it does not comply with the sustainable development objectives and lags behind the MaaS platforms in terms of costs and travel time [63]. The objective of MaaS platforms is to provide an alternative to the use of private transportation with several underlying consequences, including a reduction in traffic congestion and volume restrictions on urban transport capacity. In the coming years, we will witness the emergence of a global platform that integrates various modes of transport, with an on-demand service and where information is available in real time and in a predictive manner in such a way that allows the provision of services such as multimodal routes. Several services have appeared in recent years that promote the vision of the MaaS paradigm: carpooling (sharing the car for a given trip in order to prevent several people from traveling to the same place), ridesharing companies (companies that match passengers and vehicle drivers), bicycle and e-scooters sharing systems (systems that allow the rental of bicycles or electric scooters for a short period of time) or carsharing (car rental model for short periods of time). The recent advances in autonomous cars are a promise for the short-medium future to help materialize the MaaS paradigm. This will put in perspective the need for people to own a car, both in economic terms as well as in benefits compared to using on-demand services that are expected to have much more affordable costs when the use of autonomous vehicles becomes widespread.

One of the challenges that the coming years will bring includes the creation of a global MaaS platform integrating several geographically dispersed MaaS platforms and heterogeneous in their genesis, which could also lead to the creation of unified standards. Two main characteristics can be pointed out to a MaaS system [63,64]: (1) to be multimodal, a MaaS platform must necessarily include different types and modes of transport, and (2) to be user-centric, the result of a MaaS platform must be adapted to the needs and preferences of each user, preferably collected in an implicit and transparent way according to what is the history and patterns of their mobility.

Some of the challenges that a MaaS will help solve in a smart city include [63,64]:

- High number of private cars within a city
- Little attractiveness for citizens when it comes to using public transport
- Little use of active modes of transport
- Less adequate location of specific places for bicycles, scooters and parking links and its lack of integration with the transport network
- Lack of accessibility to transport systems by people with disabilities or old people
- Lack of information in real time for citizens
- Lack of an integrated public transport platform, routes suitable for each user, on-demand parking, active mobility and unified payment systems for the entire transport network
- Lack of understanding of citizens’ mobility patterns
- Lack of information for citizens about the consequences of their fewer active habits in the carbon scab

The benefits we can expect from a MaaS include [63,64]:

- An easier way for citizens to plan, book and pay for mobility services (which will also facilitate citizens abandoning the private vehicle)
- Improving the efficiency of the transit network
- Reduced costs for citizens
- Decreased traffic congestion
- Reducing the ecological footprint
- Predicting demands
- Making personalized suggestions to citizens
- Allowing providers to plan ahead and meet citizens’ needs
- Increasing convenience, effectiveness and customer satisfaction
- Ease of payment
- Revenue growth for transportation service providers.

#### 4.1.1. MaaS Implementation Challenges

The emergence of the smart city concept has brought a paradigm shift in urban mobility systems. Today, with technologies such as Internet of Things (IoT), Wireless Sensor Networks, Data Analytics, Big Data, etc., the transportation sector is seeing a new scope for significant changes and transformations. Today, the transport sector has become a complex system with services integrated into one platform governed by technology. MaaS describes a transport system that integrates various transport mediums along with other related services to provide a seamless on demand experience to users [65]. An efficient MaaS system provides significant potential to facilitate business and opportunities.

Today, most cities face challenges in terms of their transportation systems and other related services such as parking, traffic congestion, etc. MaaS platforms have huge potential to provide solutions to overcome the existing challenges of transportation systems and also have a positive impact on the environment by promoting and supporting renewable energy resources [66]. However, despite this utopian concept of on demand seamless mobility, there are still many challenges and issues pertaining to the successful integration of MaaS in the existing smart city ecosystems. Table 3 presents some of the major issues and challenges and the potential solutions in the implementation of MaaS platforms in a smart city ecosystem.

Multiple Services Integration: MaaS focuses on aggregating various services such as public transport, private transport, ride sharing, car pool services, payments, parking systems, etc., on a single platform to ensure on demand seamless mobility to the users. However, the heterogeneous nature of these services along with their different safety and privacy requirements makes the entire integration process difficult and challenging.

Payment System Integration: the complete and efficient implementation of MaaS platforms ensuring a seamless mobility experience requires a hassle free payment system that allows users to manage the various mobility services through a single platform. Integrating a single payment system involves multiple stakeholders at different levels of hierarchies, such as banking systems, transport companies, parking systems, vehicle owners, etc. This requires significant technological development in overcoming the security, safety and privacy requirements of multiple stakeholders and heterogeneous entities and services.

MaaS Subscription Models: currently, the existing transportation systems and companies provide their users with subscription models based on weekly, monthly and yearly services. These packages allow users to avail various services at lower costs as per the terms and conditions of the particular company. Implementing a similar service with the MaaS platform requires cooperation and coordination amongst various companies, transportation service providers, governments, private players and other related services. Ensuring a single subscription model for a MaaS platform is still a challenge.

Data and Information Sharing Amounts Service Providers: the implementation of an efficient MaaS platform requires real time data sharing amongst the various service providers to ensure a real time seamless mobility service to the users. This poses serious security and privacy concerns as many stakeholders do not wish to share their personal information, payment histories, transportation preferences and habits with others. Additionally, since MaaS integrates multiple services at different hierarchical levels, the privacy concerns and security techniques vary significantly. As per the General Data Protection Regulation (GDPR), businesses must protect the personal information of users (https://gdpr-info.eu (accessed on 3 February 2021)), and therefore MaaS platforms must also comply with the digital privacy of their users.

Legal Challenges: MaaS platforms facilitate the integration of multiple services on a single platform and also provide opportunities for new business models. The involvement of multiple services such as transport, traffic systems, parking, payments, ticketing, identity management, data sharing, etc., requires standardized laws and regulations governing the entire process. Currently, there are no legal services and laws that regulate the entire MaaS platform.

Adoption Challenges: one of the biggest challenges for the successful implementation of a MaaS platform is the willingness of the various stakeholders such as users, the government, service providers, etc., to adopt the service model. Although smart transportation systems are slowly and steadily making their way into the daily lives of the people, the mass adoption of platforms such as MaaS is still far from reality.

Scalability: the scalability of the MaaS platforms from a locality to a city, from a city to a country and eventually to the entire world requires rules that can encompass the laws pertaining to various nations. Developing a service model that can adapt to multiple cities and countries and can be reused in multiple jurisdictions is still a challenge.

Trust and Collaboration amongst Stakeholders: the need for trust is one of the most important aspects of a successful collaboration. Multiple entities in a MaaS platform require a clear understanding of the financial, societal and environmental gains of the collaboration. There are apprehensions related to the neutrality and fairness of the system from different viewpoints. From the user’s viewpoint there may be questions related to the quality of the service and value for money, and the service providers may have fears related to the manner in which the algorithms work and the services are presented to the users [67].

#### 4.1.2. Analysis of Security Threats

Autonomous vehicles use technologies such as Internet of Things, Wireless Sensor Networks, Artificial Intelligence, and Machine Learning, among others, to collect, analyze and share data and eventually make informed decisions. These autonomous vehicles form a part of a larger network comprising thousands of interconnected entities. These types of connected environments consisting of heterogeneous devices are highly dependent on the individual device’s security protocols. This makes autonomous vehicles vulnerable to several types of security threats and attacks. These attacks can take place at various levels such as the network level, device level and software level [68]. Table 4 shows the various types of vulnerabilities in autonomous vehicles at different levels.

The vulnerabilities at different levels pose serious security and privacy concerns related to the use of autonomous vehicles. Some of the major security concerns include data security, network security, vehicle security and financial security [69].

The main types of security threats include:

1. **Data Theft**: Autonomous vehicles and self-driving cars form a part of a larger network of connected entities which continuously share data to provide a seamless user experience. Some of the data generated through different entities consist of personal user information such as financial details, contact details, travel habits and history. With this type of personal data at disposal, there are vulnerabilities related to data theft that can be used by attackers.
2. **Identity Theft**: The concept of autonomous vehicles focuses on the transfer of control from the human drivers to the vehicles. In such scenarios, the connected vehicles become easy targets of cyber criminals and hackers. These vehicles are prone to identity theft where the hackers can obtain the vehicle identification information and misuse it.
3. **Device Hijacking**: Vehicle hijacking is one of the biggest threats to autonomous vehicles. The attackers can gain control of the vehicle system and software and modify the algorithms to remotely control the vehicle. Once hijacked, the hackers can modify the important functioning units such as the navigation control unit, engine, brakes, heating systems, communication systems, vehicle camera and vision systems.
4. **Denial of Service**: Attackers can take advantage of the vulnerabilities of the least secured devices on the network to gain control of the entire network and overwhelm the connected entities. These types of attacks can be launched on an individual node or vehicle-to-vehicle or vehicle-to-infrastructure, resulting in a communication system disruption.
5. **Privacy Infringement**: Autonomous vehicles continuously generate data such as the vehicle location. These data provide personal information related to the user’s travel history and navigation, which can be misused to track the user’s current location and other whereabouts.
6. **Financial Fraud**: In countries such as India where toll taxes are automatically deducted using RFID tags on the vehicles, there are serious concerns related to the financial security of the linked bank accounts. Any insecure network or device may result in banking frauds and other related attacks such as ransomware.

### 4.2. Traffic Flow Optimization

The continuous growth of traffic is a problem that many cities face today, as a result of planning that in many cases was carried out many decades ago, when the congestion experienced today was not expected. Population growth, which is taking place worldwide, has had a direct impact on the number of vehicles on the roads without proper monitoring of infrastructures as they cannot adapt as quickly as what would be necessary to avoid the problem of traffic congestion [70]. This high number of vehicles that resulted in major traffic congestion has also resulted in excessive fuel consumption and gas emissions and an increase in the number of accidents. All these aspects have a direct impact on several dimensions such as economic, environmental and living [71,72]. The US Department of Transportation (DoT) [73] points out three main sources for traffic congestion: (1) events that somehow influence traffic, such as accidents, working areas or bad weather conditions; (2) fluctuations in the traffic demand, which are the cause of not having a similar and constant pattern, and (3) existing infrastructure, which includes traffic control devices and also physical bottlenecks. Traffic Management Systems (TMSs) are the systems responsible for preventing traffic congestion and improving traffic efficiency. To this end, one of their components is the collection of information from various heterogeneous sources such as vehicles, traffic lights and sensors placed in different locations of the road infrastructure. The processing of these data is another vital component of a TMS with the purpose of predicting potential problems in a predictive or even real-time manner and acting in a timely manner to reduce traffic congestion and all related problems [70]. From the various proposals in the literature regarding TMSs, the following contributions can be enumerated: speed adjustment of the vehicle so that it spends the shortest possible time stopped at a traffic light [74,75], the detection and prevention of traffic congestion [76,77] or recommendation for alternative routes [71,77,78].

Some of the challenges being faced regarding traffic flow optimization include [48]:

- Optimization of the use of road infrastructure
- Integration of different domains (air, land and sea)
- Conducting predictive traffic analysis, with emphasis on moments when a greater flow of vehicles is expected
- Performing data analysis that allows traffic planning and control in real time
- Optimizing the location of electric car charging stations to the places where they are most in demand
- Developing predictive models that understand the needs of citizens in terms of new mobility models (including e-scooters, e-bikes, carsharing, carpooling).

According to [2], Traffic Control and Optimization will be achieved in the coming years through a network of intelligent sensors, location-based applications and an intelligent infrastructure that, acting in an integrated manner, can contribute to making traffic management, driving and even parking more efficient. ICT solutions will be the fundamental basis for controlling and optimizing traffic, allowing, for example, collision alarm and lane-keeping-systems and also integrating private vehicles with smart streets, traffic lights and TMSs. This type of solution and integration will contribute to the safety and convenience of all stakeholders. From the moment that cars, streets, traffic lights and control systems are interconnected, real-time data analysis will be possible and will allow traffic conditions to be analyzed and preventive action to be taken. Important information will then be possible to be provided to users to optimize their routes in terms of time and in order to avoid congestion or accidents on the road, adjustments to be made to the speed of their driving or information on nearby parks or parking spaces. This integration and the possibility of real-time access to information and instant communication with drivers will be reflected in terms of safety, less accidents, greater efficiency in driving and planning routes and reducing congestion, as well as the emission of gases.

### 4.3. Optimization of Logistics

Urban mobility and the current challenges we face are not just about the mobility of people and private vehicles. We must also take into account the mobility of goods, commercial transport and urban logistics. These aspects are also important to achieve sustainable and environmentally friendly mobility. If we previously referred to traffic congestion with a greater focus on private vehicles, we must also consider the impact that commercial fleets have on traffic congestion. E-commerce is nowadays a global reality with an increasing tendency of use even for the ease it brings to the consumer. However, this brings the need for deliveries, which has a major impact on urban logistics and also traffic congestion. Another aspect is restaurants and home deliveries, which started to increase in size with Uber-Eats and other similar services [79]. The mobility aspect associated with businesses is thus something that deserves as much concern as private mobility. At the present time, logistics operations are very defragmented, with little integration between the various players, making inefficient use throughout the supply chain. These problems will tend to worsen as the need for the delivery of goods increases, which is expected to be almost certain given the increase in e-commerce businesses, home meal deliveries, among others. As with the increase in the number of private vehicles, the increase in the number of deliveries and logistics processes will also have significant consequences for the increase in fuel, energy and material costs, making the need to optimize these processes obvious [2].

Some of the challenges being faced regarding traffic flow optimization include [63]:

- Vehicles that deliver not being fully filled in terms of their transport capacity
- Inefficient route optimization to make deliveries
- Lack of coordination between transport providers in order to achieve an integrated way that includes several fleets
- Little awareness by consumers and suppliers of the carbon footprint implications of the large number of deliveries
- Collision of the delivery times with the hours of greatest traffic congestion in the cities
- Deliveries made using vehicles that use fossil fuels
- Inefficient coordination of the different means of transport (sea, air and land)
- Use of private transport by citizens when shopping, instead of using public transport.

According to [2], smart logistics is related to the integration of vehicles, products and load units that allow route and load optimization, thus reducing waste in the system as a whole. The current data and sensor processing technology will allow the creation of solutions that aim to increase the flexibility and efficiency of road, air, train and marine freight through the integration of the various players in the logistic process that includes individual vehicles, roads and load units. It is expected that platforms for fleet management and optimized route calculation will allow increased efficiency in the operationalization of logistics systems as well as the planning involved in the process, reducing costs, transport with loads that do not make use of the full capacity of the vehicle, accidents or even damage to the products. Sensor technology can contribute to the monitoring and tracking of the location of products and fleets, which allows real-time changes to routes whenever necessary to avoid congestion and reduce time.

### 4.4. Autonomous Vehicles

The automation and robotization of tasks, with the increasing availability of new sensors and software, is something we currently assist in several domains, from industry to call centers, applicational support to clients, among many others. One of the applications of automation that we can call the most ambitious is the Autonomous Vehicle (AV). If we classify AVs as ambitious it is because they involve people and because, in some cases, we may be talking about situations in which their physical integrity is at stake and even life or death situations. The availability of AVs is undeniably a growing trend, and today we are witnessing an exponential evolution until a few years from now we can reach the complete automation of vehicles. The technologies that are present in cities today allow us to anticipate that in the coming years we will have frank developments regarding the automation of vehicles for the daily commuting that all citizens need to undertake for their daily tasks [80]. This will undoubtedly revolutionize urban mobility as we know it in a very drastic way. The infrastructures of cities will have to undergo significant changes in order to adapt to this new reality. The advent of 5G will be a fundamental piece of this puzzle, with a speed 100 times faster than 4G [81]. The Automotive Vehicle Readiness Index refers to the Netherlands as the country most prepared, in terms of infrastructure, to accommodate future AVs, followed by Singapore and the United Kingdom. Three main characteristics can be referred to as fundamental when it comes to changing infrastructure in cities [81]:

- Lane marking: these markings are not the best for today’s vehicles. It is an aspect that will necessarily have to be improved to the point that they can be read efficiently by machines.
- Roadside sensors: this type of sensor should be included in sidewalks, curbs and lanes. In this way, it will be possible for AVs to be aware of the environment that surrounds them and thus act in a preventive way to possible situations of danger.
- Smart signage: image recognition is currently used to read traffic signs. In the future, it is expected that the signals will be able to send a signal that can be read by machines and thus facilitate the reading of the signals by the autonomous vehicles.

The introduction of AVs is considered to have the following consequences in cities [82]:

- Providing a safer and more reliable means of transport,
- Reducing the number of accidents,
- Reducing the need for human intervention during driving,
- Reducing traffic congestion
- Allowing elderly people or people with disabilities to make their lives easier
- Elimination of traffic lights as autonomous vehicles will be able to efficiently set priorities
- Reducing/eliminating the time spent searching for parking spaces.

On another hand, some of the challenges facing the widespread adoption of AVs include [43,82,83,84]:

- Perception, planning, control and ethics
- Data privacy
- Transmission security
- Processing latency
- Energy efficiency.

#### 4.4.1. Levels of Autonomous Driving

**Figure 7:**
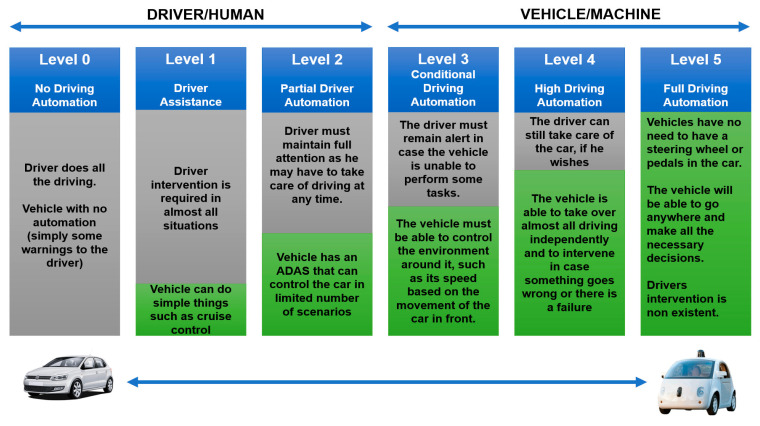
Level of automation of autonomous vehicles.

The Society of Automotive Engineers [85] defined six levels of vehicle automation which were adopted by the U.S. Department of Transportation. The levels range from 0 (fully manual) to 5 (fully automated), as described in Figure 7.

Level 0 (Fully Manual—No Driving Automation): most vehicles today are at this level where they are fully controlled by the driver. There may be some driver support systems, such as an emergency braking system and some type of warning to the driver, but that is not considered automation.

Level 1 (Driver Assistance): this level is characterized by the existence of some type of autonomous action on the part of the vehicle, such as accelerating or braking (cruise control), but driver intervention is still required in most situations.

Level 2 (Partial Driver Automation): this level is essentially characterized by the existence of Autonomous Advanced Driving Assistants (ADAS) that control the acceleration and braking performed by the vehicle, in limited scenarios. The level of automation is still low as the driver must maintain full attention as they may have to take care of driving at any time.

Level 3 (Conditional Driving Automation): level 3 represents a substantial technological advance since the vehicle must be able to control the environment around it, such as its speed based on the movement of the car in front. Even so, the driver must remain alert in case the vehicle is unable to perform some tasks.

Level 4 (High Driving Automation): the main feature introduced at level 4 is the ability of the vehicle to be able to take over almost all driving independently and to intervene in case something goes wrong or there is a failure. In most cases, driver intervention is not necessary, but the driver can still take care of the car, if they wish.

Level 5 (Full Driving Automation): level 5 represents full vehicle automation where there is no need to have a steering wheel or pedals in the car. The vehicle will be able to go anywhere and make all the necessary decisions.

#### 4.4.2. Vehicle Sensors

Sensors will play a key role in asserting AVs and in the confidence that citizens will have in these vehicles and the safety they can offer. The sensors will be an essential part of vehicle-to-vehicle (V2V) and vehicle-to-infrastructure (V2I) communication and will feed Advanced Driving Assistance Systems (ADAS) in the recommendations given to drivers. Examples of these recommendations include warnings for the driver to stay in the central part of the road, avoiding drowsiness or distraction when managing cruise control, or in more complex situations that cross information from the outside weather with the driver’s condition (calmer, more aggressive), or with their heartbeat, which are among many other aspects that can be monitored in order to collect as much information as possible to create a safe driving environment that drastically reduces the number of accidents.

In order to collect the information mentioned above, it is necessary to have several sensors in the vehicles, namely environment sensors which are responsible for collecting information from the environment surrounding the vehicle [86]. Within the environmental sensors, there are cameras and remote radars placed in front of the vehicle, long-range radars that allow the detection of objects in front of the vehicle when it is at high speed and Lidar Laser Scanners that allow the detection of objects at medium distances. It is normal for each of these sensors to be placed in different locations on the vehicle because of the effects of time, the detection angle and the maximum distance at which an object is identified. Table 5 summarizes the vehicle’s main sensors, location and their purpose.

#### 4.4.3. Vehicle Communication Protocols

Vehicular Ad-hoc Networks (VANETs) play a key role in intelligent transport systems (ITS). In VANETs, the development of a routing protocol for effective vehicular communications presents several challenges, which can be highlighted [87]:

- Safe routing: the security of messages is, in all systems, delicate and of enormous importance. In this specific case, illegal message tampering can have very serious consequences and even have an impact on life-or-death situations;
- Reliable communication: link ruptures can suddenly happen due to some VANETs’ characteristics such as high mobility, intermittent connectivity or obstacles in cities, and what to do in case of packet losses is a challenge;
- Determining the optimal path from a source to a destination, taking into account the density of traffic and the shortest distance;
- Determining a routing strategy that adapts to the two distinct environments of VANETs: city environment and motorways.

**Figure 8:**
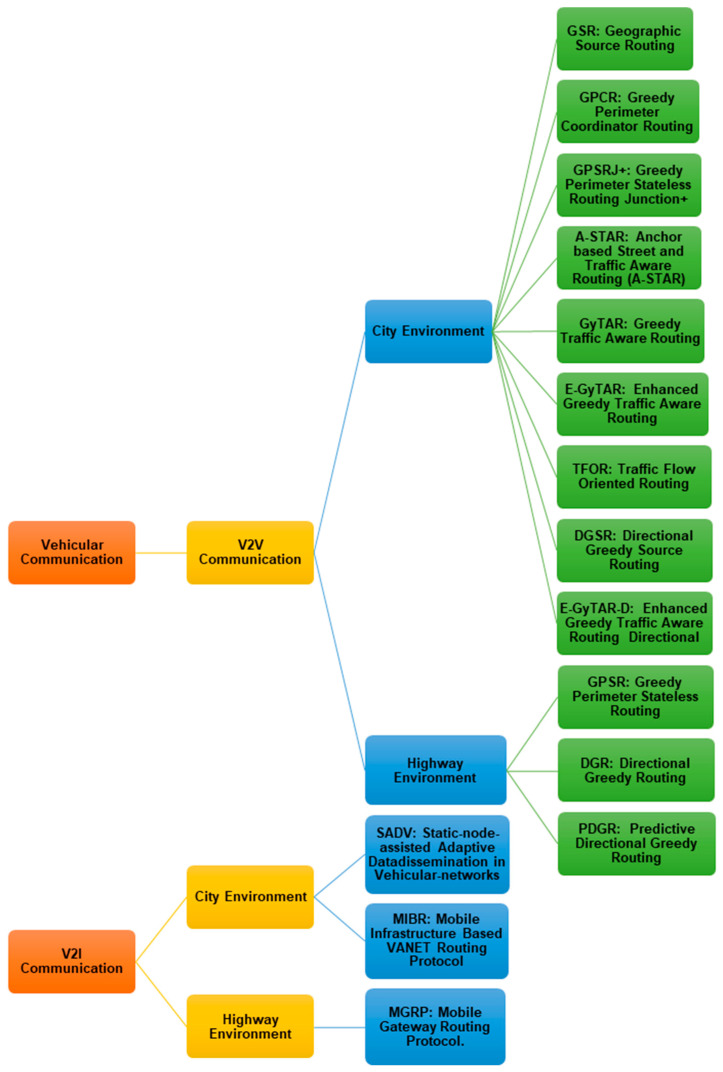
Categories of protocols on vehicular communications.

The position-based routing protocol is considered one of the best approaches in VANETs [87] and can be adopted in an urban environment (where the biggest challenge is the obstacles) as well as on highways. In the literature and taking into account the challenges of routing in an urban environment, there are many proposals for the adoption of this type of protocol for communications V2V and V2I [88,89,90,91,92], with the various categories of protocols listed in Figure 8.

#### 4.4.4. Ethical Issues

The recent popularity of autonomous vehicles has drawn the attention of automobile manufacturers from all over the world. Autonomous vehicles represent any means of transportation that are capable of navigating the roads with no or minimum human interventions and provide potential solutions to the road safety issues caused by human errors [93]. The transition from a purely human operated vehicle to a fully automated vehicle is still far from reality. However, many automobile companies are already offering partial automation in terms of services such as emergency brakes application, assisted parking, etc. Despite the several benefits and the rising popularity, the deployment of these vehicles on the road poses several critical challenges related to the operations, technology, legal aspects and, most of all, ethical issues [94].

The main concerns regard how AVs should behave in extreme traffic situations. In the literature, this is known as the trolley problem, first proposed in 1967, where a moral choice must be taken, and the AV should decide whether to kill five people by allowing the trolley to proceed, or kill “just” one person while stopping the trolley [95]. This issue has been deeply debated as the Moral Machine Experiment (MME), but there is still no solution. From this research, it emerged that customers are willing to buy AVs that prioritize the safety of their passengers, whilst they agree on others buying AVs that sacrifice passengers’ life for a greater good [96]. This is no more a technological problem, but Philosophy, Law and Ethics are involved and the individual good is opposed to a greater one [97]. The trolley problem was the first example of extreme traffic situations that lead to a moral choice. Several situations have been discussed in the literature. Just to mention a few, one regards whether to kill a child or an elderly person to avoid an obstacle, or whether to invade the opposite carriageway, thus involving more cars in an accident, to avoid collision with a heavy goods vehicle that surely would strongly injure the AV [98]. From a technical point of view, these choices can be addressed by rules that embed the human driver experience and return risk and gain probabilities. However, first of all, the problem is so complex that it is not possible to foresee all possible situations; moreover, other ethical and moral issues arise: there is the need to judge if the rules are acceptable, then the same rules should be adopted by all the companies, and finally the main issue regards the damage responsibility [98]. It is worth noting that whilst in the scientific literature this moral dilemma has been largely debated, and yet no answers have been found, industry reports on the same topic assume a pragmatic approach. Indeed, these extreme traffic situations are considered to be rare cases that cannot be avoided, and damages to objects and people must be minimized [95]. However, with the spread of the first autonomous vehicles, some accidents have already happened. This is the case of a pedestrian who was killed in Arizona by an Uber self-driving car (https://www.bbc.com/news/technology-54175359 accessed on 3 February 2021).

Since this topic is very critical and it limits the development of AVs, recently, ethical guidelines for the automotive sectors have been released by detailing the fundamental requirements and practical recommendations for both industries and policymakers [99]. Particularly, in 2019 seven fundamental requirements were proposed by the High-level Expert Group on Artificial Intelligence, which included “human agency and oversight”, “technical robustness and safety”, “privacy and data governance, transparency”, “diversity, non-discrimination and fairness”, “societal and environmental wellbeing” and “accountability”. Then, the guidelines have pointed out that five rights underlie these requirements, namely: the right to “self-determination and liberty”, “life and security”, “protection of personal data”, “equality and nondiscrimination” and to “explanation”. Table 6 summarizes the seven requirements by highlighting their key concerns and brief descriptions. Interested readers can refer to [99] for more details.

### 4.5. Outdoor Navigation Technologies

Outdoor navigation represents an important component of one of the services that can be delivered to citizens under smart mobility. In this section, we mention some of the techniques that are being used to achieve that and serve as the basis to deliver applications and services to citizens in order to ease one of the most common tasks nowadays, namely when we are in unknown places or also when we are talking about people with permanent or temporary disabilities such as visually impaired people, autistic people or people in wheelchairs for whom navigation represents a major issue. One of the main and first technologies for outdoor navigation is the Global Positioning System. It is at the base of the guidance systems that emerged in cars, and its main limitation is its limited accuracy. GPS technology was first made available with a deliberate error—called Selected Availability (SA)—that could cause an error of 100 m for civilian usage, which highly limited applications that need to have an accurate positioning [102]. A GPS receiver is based on signals received from several non-geo-stationary satellites. The fact that the number of satellites, as well as their position, is variable directly affects the accuracy [103,104,105,106]. Even after the SA limitation was over in 2000, the accuracy achieved with only the use of GPS is limited for solutions such as high-precision agriculture or pedestrian navigation in locations with tall buildings [107]. To solve these limitations, alternatives have emerged, such as those based on differential corrections and which support methods such as RTK or DGPS, which still have the signal degraded or have interruptions depending on the range and quality of transmission [108]. DGPS can be defined as a real-time positioning method that makes use of at least two GPS receivers, one installed at a point with known coordinates (called a base or reference station) and the other moving along a given path whose coordinates need to be discovered. The base station calculates the differential corrections and transmits them to the mobile receiver so that the accuracy of its location can be improved. Another approach is the use of active beacons that can use radio, sonar or laser and determine the location of the person or object based on the triangulation of the signal. The delay in receiving the signal causes inaccuracy in the location obtained and there are also high costs in the associated infrastructure, which requires the existence of many beacons [109]. Dead reckoning is an ancient maritime term to describe navigation based on the starting position and a velocity vector composed of speed and direction and how long the velocity is maintained to determine the new position [110]. This technique, applied to pedestrian navigation, measures and analyzes a person’s way of walking in order to understand and measure displacement in relation to an initial position based on a set of sensors that allow the extraction of speed and altitude information. The calculated positioning is based entirely on the information collected through sensors [111], the error being accumulated because the current position is calculated based on the previous one, and so on. This method has been used in conjunction with GPS to improve the determination of a user’s position outdoors. Dead reckoning is also being used in terms of navigation and vehicle location [9], where it is possible to install odometer and optical sensors for wheel direction detection and achieve a low-cost solution, however, keeping the problem of error accumulation. Visual positioning is a technique that aims to overcome the imprecision of outdoor positioning obtained with previous mentioned technologies using visual elements such as markers, landmarks combined with camera images captured by a smartphone. Google is currently developing its Visual Positioning System (VPS) which is integrated with Google Maps so that, based on augmented reality, navigation takes on another dimension while using the camera to inspect the surroundings, infer position and adjust positioning and navigation recommendations [112]. This is achieved through considerable computational power combined with a large set of data on the backend that allows the recognition of the position based on the image captured by the camera of the mobile phone, mostly through buildings and tourist spots. This approach is focused on the use of known landmarks (monuments, etc.) that can therefore quickly and unambiguously, or at least with great precision, identify the user’s current position. Some of the advantages of this approach include [112,113]: higher accuracy than GPS, and therefore an alternative for locations with tall buildings, added value for blind people, whether on the streets, at a school or in an establishment, useful for situations where a person is lost without knowing their location, added value for companies, who can use this system to provide navigation information to their customers to travel to their establishment, either on foot or using public transport, the availability of application in indoor and outdoor environments, high scalability and low cost. Like all technologies, this one too has its limitations and challenges, which include difficulty, in some locations, to have landmarks that can be used to accurately identify a particular location, difficulty in mapping the interior of all buildings, vulnerability to luminosity, causing shadows with impact on the identification of places, and the possibility of people or objects being able to block the captured image and thus prevent the recognition of the location. Passive markers are being used to provide an optimized route that goes through several points of interest. The current location can be obtained through passive markers that are strategically placed near points of interests which have been previously mapped to a Spatial Map Graph, using machine learning [114]. Passive markers, in an outdoor environment, might be represented through QR codes that are placed in places such as lamp posts or trees. An example of visual landmark-based localization is reported in [115], where the authors present a study on location based on geo-referenced landmarks, whose images are collected by cameras in order to serve as an input to the location functionality that allows the attainment of the precise location of micro aerial vehicles in an urban environment. The use of vision/landmark-based positioning is also reported in [116], where the authors describe two different ways of acting: on the one hand, what they call “single snapshot-based positioning”, which consists of obtaining an image and comparing it with a set of images previously taken and georeferenced through an image matching algorithm. On the other hand, they present a scenario based on “continuous localization”, which is combined with a dead reckoning compass allowing an accuracy of 10 to 15 m.

## 5. Enabling Technologies to Support Smart Mobility

### 5.1. Overview of Enabling Technologies

**Figure 9:**
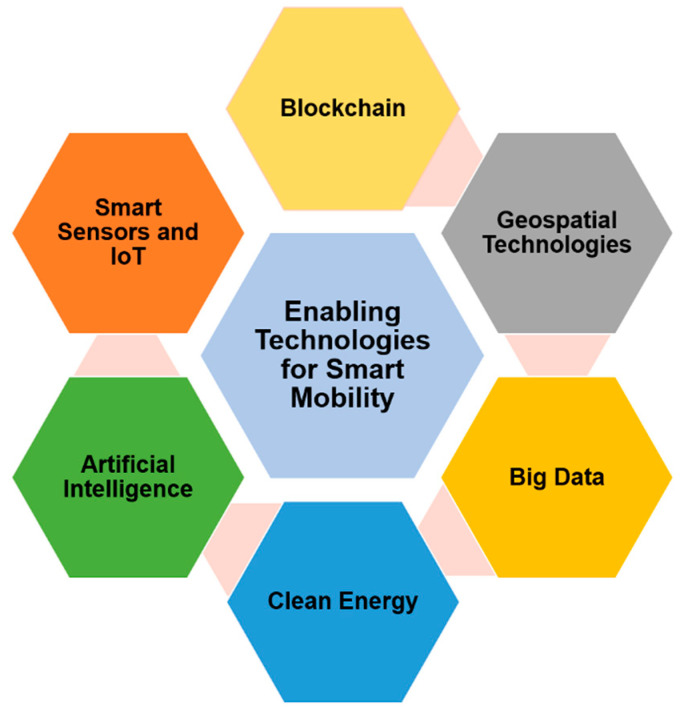
Enabling technologies to support smart mobility.

Smart mobility is a growing trend due to the advent of several technologies and concepts that, when combined, allow us to think of solutions that will effectively make a difference in the future in terms of new products and new data processing algorithms and techniques that will provide useful information to the citizens, in order to provide more autonomy and more well-being in their daily lives. There are several enabling technologies that can facilitate the smooth adoption of smart mobility. Figure 9 shows these technologies [117,118,119,120,121,122,123].

Blockchain: Blockchain technology can provide a privacy preserved, transparent and trustless architecture for mobility services for inhabitants. Blockchain based Internet of Vehicles (IoV) architecture can be created for improved interaction and communication between vehicles as well as improved tracking and management of traffic within smart cities.

Smart Sensors and IoT: Intelligent and energy efficient sensor technology can be effective for sensing and collecting real time traffic and mobility data of the vehicles as well as the inhabitants to provide effective mobility management. Smart sensor-based streetlights and traffic signals can autonomously make intelligent decisions on the basis of real time situations to facilitate the smooth management of traffic.

Artificial Intelligence (AI): On the basis of the data collected from different sources, AI based algorithms can be developed to provide autonomous decisions as well as future predictions about different mobility related services and entities, such as the conditions of roads, traffic, and streetlights. Additionally, data centric AI algorithms can be used by organizations for customer centric target marketing.

Geospatial Technologies: Intelligent geospatial technologies can provide accurate information for the tracking and tracing of vehicles and citizens. These technologies can also facilitate alternative routes management in case of emergencies and disasters.

Big Data: Big Data forms the backbone of the smart city ecosystem. The enormous amount of data generated from the sensors and IoT devices can provide valuable information about the subject and its surroundings. This information can be used to extract valuable information.

Clean Energy: Smart mobility must be complemented by clean energy alternatives. Clean energy sources such as solar energy, wind energy, hydro energy and biomass, etc., must be adopted to provide zero-emission fuels to power the smart city ecosystem.

**Figure 10:**
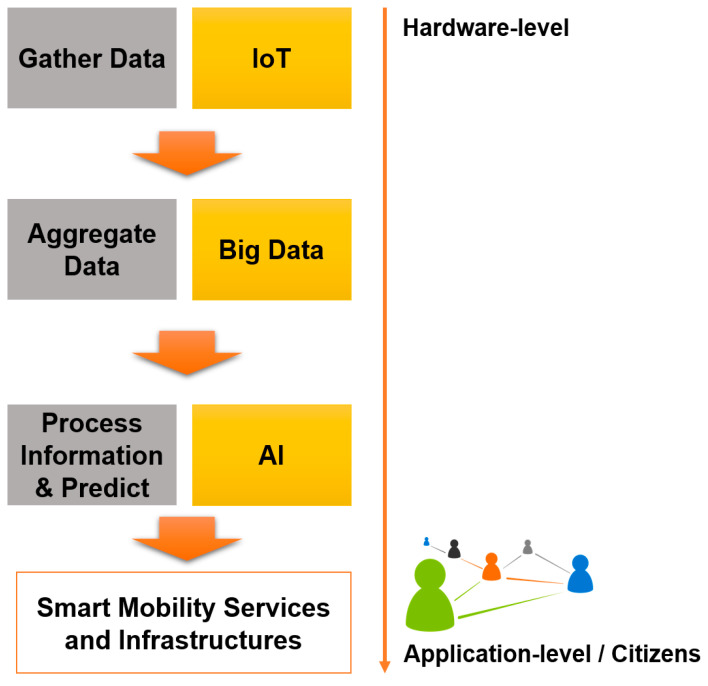
Contribution of some of the enabling technologies to smart mobility.

Figure 10 shows how some of these concepts can contribute to smart mobility services and infrastructures, starting from the hardware-level gathering data based on an IoT layer composed of several devices on multiple levels; next, an aggregation layer with Big Data and the creation of datasets with a huge amount of data; and finally, the processing of that information using AI that will also allow the prediction of trends and support decision making. All this combined allows the delivery of end services to citizens to improve mobility in smart cities.

The concept of Big Data is an emerging term that is gaining more and more emphasis given the technological evolution we are witnessing and the heterogeneity of systems and sensors that emerge and which apply to various domains. At its base, this concept is intrinsically linked to the existence of large volumes of data, collected from various sources and, therefore, heterogeneous. The relevance of Big Data only arises when information processing techniques are applied to these data so that predictive information and pattern analysis can help them in making decisions. The sources that now feed data into the datasets that make up Big Data are immense and include smartphones, data from social networks, vehicle sensors, GPS and location applications, traffic light sensors and other road locations, public agencies, connected cars, and camera feeds.

The General Data Protection Regulation may constitute an obstacle to the use and adoption of Big Data in smart mobility services. Some examples include:

- Real-time information about the location collected from smartphones that can be used for various services on people’s mobility can be a major barrier in terms of privacy;
- The collection of images of people obtained from services for mapping and recognition purposes;
- The on-board units of the vehicles might be associated with plate numbers which are considered indirect identifiers;
- The lack of verification of the accuracy and relevance of the information collected.

Big Data, combined with AI techniques, can allow significant improvements in several aspects and dimensions of smart mobility [124]:

- Port scales can be optimized if data are shared in advance in order to improve planning and resource allocation. In Hamburg, for example, electric vehicles and real-time navigation are used to ensure the smooth flow of traffic and thus reduce congestion;
- Traffic lights can be controlled based on traffic flow if the necessary data are collected. In this way, traffic in cities can be optimized and congestion can be reduced. This is an example currently in practice in the city of Hong Kong;
- IoT sensors and CCTV cameras can also be used for traffic management, thus reducing congestion;
- Open data about mobility can be of added value to citizens that can be aware of real time traffic data and plan their day with more information so it can be more efficient;
- Parking spaces can be monitored, and drivers can reduce parking time and also CO_2_ emissions;
- On-demand and more adaptive modes of capacity planning and operations can be achieved using data from sensors, cameras and vehicles so that information to commuters can be given so they can plan their routes more efficiently;
- Fleet efficiency can be obtained by combining real-time traffic data to produce route optimization using AI techniques, reducing wait times and optimizing energy consumption;
- Increase citizens’ security by preventing anomaly detection and anticipating incidents.

### 5.2. Role of Enabling Technologies in Smart Mobility Services and Applications

In this section, an analysis is made of the current use of enabling technologies in the main services and applications in the area of smart mobility as previously discussed in Section 4.

#### 5.2.1. Role in Mobility-as-a-Service

In MaaS systems, a central operator made available through an intermediate layer of the system becomes essential to manage all communication between transport companies, passengers and other stakeholders of the system. In [125], the authors propose a blockchain-based solution that eliminates this intermediate layer, promoting trust and transparency among all stakeholders and eliminating the need for commercial agreements between the various MaaS agents. In [126], easy, quick and trusted transactions are covered, using AI and blockchain-enabled smart contracts.

The use of geospatial technologies has big applicability in MaaS solutions, bringing added value to the entire system and associated services. One example is the monitoring of commuters. In [127], the authors exploit Multi-access Edge Computing (MEC) to propose a MaaS solution that collects and processes data related to the mobility of commuters in order to be able to recommend optimized routes taking into account the needs and preferences of each commuter. Another study [128] also uses MEC, IoT messaging protocols and virtualization (i.e., digital twins) in the proposal of a framework that collects data about commuters in order to feed the monitoring of mobility by the transport planning solutions.

The use of Big Data in MaaS systems is present in the aforementioned solutions that collect and process data from commuters [127,128]. A specific big data architecture is presented in [129], motivated by the specific requirements of mobility analytics.

With regard to clean energy, the entire concept of MaaS recommends the progressive transition from private to public and shared transport, including car-pooling and car-sharing. This paradigm shift will bring with it a consequent reduction in vehicles and the emission of CO_2_ into the atmosphere. In [130], the authors present a study of when the transition to MaaS could happen on a larger scale and what impact it will have on the choice of forms of mobility.

#### 5.2.2. Role in Traffic Flow Optimization and Optimization of Logistics

Traffic optimization is one of the primary needs in terms of mobility. In [131], the authors propose a blockchain-based approach focused on air traffic optimization, where blockchain technology is used to ensure a secure, transparent and decentralized platform.

The transport sector’s contribution to CO_2_ emissions is 23%, with traffic congestion accounting for ¾ of this value [132], hence the optimization of the traffic flow (private and also from the point of view of logistics) is essential if CO_2_ emissions are to be reduced.

Real-time analysis of traffic information is essential to efficiently manage urban traffic. In [133], the authors propose the monitoring and analysis of traffic through unmanned aerial vehicles (UAV) for real-time video collection, and artificial intelligence techniques for the processing and identification of moving objects. In [134], the authors propose a model for collecting and analyzing traffic patterns, using cameras at intersections in order to help reduce traffic congestion. In order to reduce network congestion, the authors propose processing at the edge, without sending the Big Data set to the cloud. Processing is performed using deep learning techniques and algorithms. Is it important to highlight that these types of approaches can also help to solve problems related to transportation more related to the logistics sector.

The use of sensors and an IoT network for traffic monitoring and optimization is the result of numerous contributions in the literature [135]. In [136], a solution is proposed to optimize the traffic flow at intersections based on ultrasonic sensors integrated with a Raspberry Pi that operate on the road lines, taking into account the density of the traffic. The authors stress the importance of using IoT to drive for smarter safety and safer devices on the road.

The performance of the various stakeholders of the supply chains, including transport logistics, needs transparency so that it can be a connected, intelligent and efficient system. In [137], the authors propose a new approach to the use of blockchain to ensure the integrity of the performance monitoring of the logistics sector. On the other hand, in [138], and given the lack of a traceability mechanism in the logistical transaction, as well as the integrity and security of the information, the authors propose a traceability algorithm in logistical transactions based on blockchain.

#### 5.2.3. Role in Autonomous Vehicles

The adoption of blockchain in autonomous vehicles already has several proposals in the literature. One of its applications is to support a system that stores events that happened in the autonomous vehicle and that can be useful when there is an accident between vehicles or involving a human being [139]. In these situations, responsibility must be decided on the basis of a system that makes it possible to understand the various events that have taken place in the autonomous vehicle. Another application of the blockchain in AVs is related to car-sharing or car-pooling and the guarantee of trust that is intended to exist among everyone involved in this type of system, which is also directly linked to the concept of MaaS [140]. Blockchain and smart contracts are also being used to ensure the authenticity and integrity of the firmware update process by manufacturers of the systems that support AVs, which are increasingly subject to attacks [141].

Geospatial technologies aim at tracking and tracing vehicles and pedestrians, thus providing a range of mobility services. In [142], a system is presented that tracks and monitors the autonomous vehicle in real time using a Frequency Modulated Continuous Wave (FMCW) radar. In [143], the authors propose a model to address the accuracy of tracking and vehicle stability. The model aims to perform the predictive control that intends to monitor any deviations in the trajectory in order to keep the vehicle stable, even at high speed.

One of the relevant aspects with regard to AVs is the choice of optimal trajectory. This choice, in [144], is made by the authors using Big Data mining and the analysis of real accident data as well as real-time data from connected vehicles. In [145], the authors propose an autonomous accident detection system in real time based on computational intelligence techniques. The study uses Big Data processing methodologies to analyze 2015 traffic flow data from Istanbul collected from different sensors, which are also intrinsically related to how autonomous vehicles are connected to the Internet of Things.

Autonomous vehicles represent the future, in which vehicles are expected to be connected and electric. This evolution is directly linked to climate and environmental sustainability, taking into account an EU directive that requires a 40% reduction in CO_2_ by 2030 compared to 1990. The reduction in CO_2_ emissions from vehicles goes largely through the transition for electric vehicles [146].

Vision-based artificial intelligence in autonomous vehicles allows a set of features including the detection of obstacles as well as mechanisms to avoid them [147]. In [148], the authors propose a way to increase the processing power to estimate the distance to obstacles using Compute Unified Device Architecture (CUDA) along with Believe Propagation Algorithm (BPA). The use of AI for in-vehicle vision systems to power Advanced Driving Assistant Systems (ADAS) is explored in [149].

Table 7 summarizes, for an easier understanding, the studies that apply each one of the technologies to the smart mobility service or application.

## 6. Future Trends on Smart Urban Mobility

Smart mobility solutions are catalyzing a paradigm shift in the area of mobility management in smart cities. Several innovative approaches are being devised to improve the state-of-the-art of smart mobility. A few important factors to be considered include:

- Mobility in degraded vision
- Electric vehicles
- Alternate fuels
- Mobility solutions in natural calamities and disasters
- Mobility for differently abled citizens
- Inclusive, environment friendly, sustainable and efficient transportation
- IoT based dynamic traffic management
- Transparent and distributed traffic management
- Security of citizens, devices and vehicles.

Artificial Intelligence is one of the pioneering technologies being exploited in recent years to provide state-of-the-art mobility solutions. Deep learning-based object detection and identification can be used to provide improved visibility in conditions of degraded vision (such as low light areas, foggy, windy or rainy environments) [150,151,152]. IoT-based road condition monitoring, environment monitoring, and surrounding monitoring can provide unprecedented information which can be used to predict future conditions to aid in the desired course of actions. Blockchain technology can provide a distributed, transparent, and immutable Vehicle-to-Everything (V2X) network for effective, secured and privacy preserved management of the smart mobility ecosystem [153,154,155,156]. Technology for low carbon emissions, alternative fuels, electric vehicles and improved batteries can also aid in the protection of the environment and attaining sustainable development goals for better environment for a better quality of life (QoL). Extensive research was conducted in the field of communications to provide connectivity in times of natural calamity and disasters [157,158]. Affordable satellite-based communication technologies are being explored to provide connectivity in extreme and unavoidable conditions.

## 7. Conclusions

The last few years have already made some advances and the first steps towards the construction of future smart cities known, namely in terms of mobility. The increase in the world population inevitably has considerable consequences on the way urban mobility operates, not least because cities were planned decades or even hundreds of years ago, taking into account a much smaller population and where infrastructure needs were very different. A challenge now arises in adapting cities to current needs. However, the evolution towards cities and smart mobility will not involve making more space for vehicles or more roads; rather, it will undergo a cultural paradigm shift in which people will have to stop using their private cars to switch to shared transport, which may thus reduce not only the number of vehicles in circulation, but also contribute to reducing the ecological footprint. In addition to the technological issue associated with this change, we also have, and in a very marked way, an underlying cultural change because there is a certain comfort and facilitation in the use of our own cars that will not be easy to change from one moment to the next. Citizens must realize the negative consequences for the environment of maintaining the current state and the impossibility of managing so many cars within the cities with the highest population density, and while there must be an effort to raise awareness of the benefits, we will all gain from this cultural and paradigm shift.

Technology and its exponential evolution, which is expected to continue in the coming years, will support the transition from Smart Mobility to a Mobility-as-a-Service paradigm, where vehicle sharing, carsharing, and carpooling will bring the emergence of a new dimension. The IoT and the connectivity that will be able to be established between vehicles, traffic lights, and pedestrians, among others, will allow the evolution to intelligent traffic management systems with reduced congestion and even the number of accidents, which is the main premise that Level 5 Autonomous Vehicles intend to achieve. To this end, we will have the big contribution of Big Data and AI that, together, will process gigantic volumes of information collected from the IoT and will allow services to be made available to citizens that will make their daily lives much easier and will allow preventive action in decision-making with regard to the management of vehicle, fleet and pedestrian traffic.

## Figures and Tables

**Table 1:** Summary of literature review.

S. No.	Paper (Year)	Focus
1.	Amoretti, M. et al. (2017)	Smart mobility application “UTravel” based on “Universal Profiling and Recommendation (UPR)”.
2.	Belbachir, A. et al. (2019)	Traffic control system based on cooperative agents.
3.	Benevolo, C. et al. (2016)	Novel action taxonomy involving a systematic approach to smart mobility and analysis of the role of ICT.
4.	Mirri, S. et al. (2016)	Smart Mobility for All (SMAll)—Framework for developing and implementing smart mobility applications.
5.	Lopez, D. et al. (2020)	Six-layer blockchain architecture to handle the issues regarding security, privacy, scalability and management of “Smart Mobility Data-market (BSMD)”.
6.	Bravo, Y. et al. (2016)	Framework “HITUL” for assistance in the decision making of traffic control management.
7.	Aletà, N. et al. (2017)	Spectrum of development of “Spanish Smart City” measures with a view to mobility and environmental concerns.
8.	Camero, A. et al. (2018)	Prediction of the occupancy rate of car parking space premised on deep learning with “Recurrent Neural Networks (RNNs)”.
9.	Cledou, G. et al. (2018)	Taxonomy for the formulation of smart city services.
10.	Dameri, R. P. (2017)	Novel action taxonomy concerning an extensive methodology related to Smart Mobility.
11.	Bucchiarone, A. (2019)	Collective Adaptation Engine (CAE)—Distributed adaptation method for ensemble-based systems in smart mobility context.
12.	Del Vecchio, P. et al. (2019)	Application of system dynamics to simulate substitutes for conventional human mobility.
13.	Debnath, A. K. et al. (2014)	Realistic structure to design a comparative analysis, which gauges cities based on the smartness of their transport frameworks.
14.	Docherty, I. et al. (2018)	Analysis of the likely transition of present issues of mobility governance with the aim to safeguard and increase the public value.
15.	Torres-Sospedra, J. et al. (2015)	“SmartUJI APP” and “SmartUJI AR” to enhance the positioning element of smart mobility in a smart university setting acting as a representative for a smart city.
16.	Garau, C. et al. (2015)	Mobility indicators to assess smart urban mobility in Italian cities.
17.	Groth, S. (2019)	Transformation from an automobile community to a multimodal community fostered by the advent of smart mobility powered by ICT in a German region.
18.	Battarra, R. et al. (2018)	Assessment of the possibility and degree of applying the smart city model with the aim to boost the effectiveness and living conditions of urban areas.
19.	Ilarri, S. et al. (2015)	SemanticMOVE—distributed system for mobility data management and semantic improvement.
20.	Papa, E. et al. (2015)	Multi-disciplinary and collective methodology to smart mobility to enable the shift to a “smarter mobility” to improve the city development and citizens’ quality of life.
21.	Jeekel, H. (2017)	Relationship between smart mobility and social sustainability.
22.	Kronsell, A. et al. (2020)	Theoretical assessment of experimental governance with respect to smart mobility in Sweden.
23.	Garau, C. et al. (2016)	Quantitative approach for assessing urban mobility in the Italian area of Cagliari.
24.	Kudo, H. (2016)	Framework of citizen engagement and efficiency of Japanese Smart Communities to contemplate the collaborative design and development of a smart mobility framework.
25.	Longo, A. et al. (2019)	Holistic methodology to model the efficiency of public transport facilities schemed as a whole in a multi-stakeholder context from end-to-end perspective.
26.	Lyons, G. (2018)	Analysis of the meaning of “smart” in the context of smart urban mobility and the relationship between smartness and sustainability.
27.	Mangiaracina, R. et al. (2017)	Analysis of the role played by “Intelligent Transport Systems (ITS)” in assisting urban smart mobility.
28.	Mboup, G. (2017)	Highlighting the factors that link citizens to different facilities, especially mobility and ICT frameworks in the Senegalese city of Dakar.
29.	Melo, S. et al. (2017)	Case study in the Portuguese city of Lisbon which establishes a performance assessment of passenger and commercial vehicle redirection.
30.	Ning, Z. et al. (2017)	Concept of “Vehicular Social Networks (VSNs)” focusing on the importance of highly efficient and secure smart city transmission in VSNs.
31.	Orlowski, A. et al. (2019)	Establishing an indicator for assessing the degree of smart mobility solutions deployed in urban areas.
32.	Pangbourne, K. et al. (2018)	Analysis of “Mobility as a Service (MaaS)” to evaluate its potential impact for city policymakers with regards to governance and sustainability.
33.	Peprah, C. et al. (2019)	Evaluating the smartness of transport systems in the cities of Ghana and illustrating the realization of the notion.
34.	Pereira, J. et al. (2019)	General framework for the implementation of fog computing features in “Vehicular ad-hoc Networks (VANET)” context.
35.	Schlingensiepen, J. et al. (2016)	“Autonomic transport management system”—ICT based system for the management of transport.
36.	Faria, R. et al. (2017)	Analysis of existing IoT methods and notions concerning smart cities and smart mobility.
37.	Turetken, O. et al. (2019)	Implementation of “Service-Dominant Business Model Radar (SDBM/R)” in the context of smart mobility.
38.	Yigitcanlar, T. et al. (2019)	Correlation between urban intelligence and sustainable mobility forms for the municipalities in Australia.
39.	Zawieska, J. et al. (2018)	Analysis of the association between the deployment of the smart city notion and the sustainable mobility notion.
40.	Singh, Y. J. (2020)	Notion of shared mobility focusing on gender parity.

**Table 2:** Smart mobility attributes.

Attributes	Significance
Flexibility	Allows users to choose from the multiple modes of transportation to suit their needs using smart and dynamic navigation.
Efficiency	Provides efficient mobility options with minimum disruptions, low cost and minimum commute time.
Integration	Ensures end-to-end route plans independent of the transportation modes.
Sustainability	Promotes cleaner and sustainable operations with minimum emissions.
Security and Safety	The efficient data sharing and connectivity models ensure road safety.
Social Benefits	Provides equal opportunities to citizens to use public transport. Ensuring quality of life to all.
Automation	Facilitates automation in all processes.
Connectivity	The entities in the network are connected.
Accessibility	Affordable to all.
User Experience	The efficient processes ensure a better user experience.

**Table 3:** Mobility as a Service (MaaS) implementation challenges and potential solutions.

S. No.	Challenges	Solution
1	Multiple Services integration	Initiating goal directed discussions amongst the various service providers to ensure cross border integrations.
2	Payment System	Technological interventions based on emerging technologies such as blockchain, can be used to ensure the security and transparency of the financial systems.
3	Subscription Models in MaaS	Government and other regulating bodies must promote collaborations through various pilot programs. Customized subscription models need to be developed to enhance the user experience.
4	Data and information sharing amongst service providers	Data sharing models can be developed regulating the general principles of data and information sharing and ensuring agreements on the levels of data sharing between service providers.
5	Legal Challenges	There should be globally recognized standards and laws governing the various services provided under the MaaS Platform.
6	Adoption Challenges	The MaaS platform developers must take measures to build trust amongst the users and create awareness about the financial, societal and environmental benefits of MaaS.
7	Scalability	International governmental bodies must collaborate for the development of laws and standards governing the mobility services and to facilitate joint ventures across cities and nations.
8	Trust and Collaboration amongst stakeholders	The governing bodies should ensure transparency in the processes by drafting well-defined liabilities and mutual contractual agreements documenting the agreed-upon actions and objectives.

**Table 4:** Vulnerabilities in Autonomous Vehicles [68].

Levels	Target Systems	Disrupted Services
**Sensors**	Camera, GPS, LiDAR, Radar, proximity sensors, ultrasonic sensors	Parking assistance, object identification, navigation, collision avoidance, traffic signal identification, cruise control
**Device**	Access control systems	Anti-theft system, keyless entry systems, signal jamming, replay attack
**Software**	In Vehicle protocols: LIN, CAN, FlexRay	Communication system

**Table 5:** Vehicle sensors, location and its purpose.

Sensor	Location	Purpose
Long-Range Radar	Front-center of the vehicle	Emergency Braking Pedestrian Detection Collision Avoidance Adaptative Cruise Control
LIDAR	Front, back and 4 corners (diagonals)	Environment mapping
Camera	Front, back and 2 sides	Traffic Sign Recognition Lane Departure Warning Surround View Digital Side Mirror Rear View Mirror
Short-Medium Range Radar	Front, back and rear corners	Cross Traffic Alert Rear Collision Warning

**Table 6:** Requirements for autonomous vehicles based on fundamental rights.

Requirements	Key Concerns	Description
Human agency and oversight	Which level of autonomy should be allowed? Is the state of the driver suitable for obtaining control? How can pedestrians and people outside the AV exercise their autonomy?	AVs must allow a level of autonomy to the human drivers. They should be allowed to override the decision of the machine intelligence. Autonomy requires drivers to be informed. On the other hand, AVs should monitor the driver’s state and block the autonomy if their state could cause risks (e.g., they are drunk, sleepy, etc.). Finally, humans outside the vehicle should also be able to preserve their autonomy.
Technical robustness and safety	How to protect AV by cyber-attacks that affect vehicle security? How to automatically manage emergency situations?	AVs must be robust to external cybersecurity attacks (e.g., Hijacking, Abuse, Passive behavioral attacks). Data communications between vehicles and servers must not reveal private data that could affect the behavior of the vehicle. Moreover, AVs must provide an “automatic safe condition state” for managing emergency situations and minimizing the risks.
Privacy and data governance	Which kind of data are stored? What is their aim? Is the driver correctly informed about data storage? Did they explicitly express their consent? Are data laws respected?	Autonomous vehicles continuously capture information to enhance their artificial intelligence systems. These data contain information related to user behavior and travel history. Sharing the personal user data must comply with the GDPR. These personal data are further vulnerable to thefts and misuse. Moreover, the type of the information collected from the AVs influences their capabilities. Thus, the kind of data and their scope must be specified (e.g., geolocalisation data for navigation, biometric data for user recognition and driver’s state evaluation, driver behavioral data for analysis). Data storage leads to the following legal issues: transparency (privacy policies must clearly describe which data are collected and why), explicit consent, sharing with third parties, compliance with data protection standards and regulations.
Transparency	Clear description of technical information behind the AV Explainability	Transparency is strictly related to privacy and data governance. The manufacturers must provide information about data collection and their use. Moreover drivers must be aware of the AV’s mechanisms for accountability. Transparency also regards the right to explanation. Drivers need to trust AVs, thus transparency and communication of the underlying functionality must be clearly explained in a way humans can understand. Explainable Artificial Intelligence studies these aspects that are crucial in smart cities where humans and machines continuously interact [100]
Diversity, non-discrimination and fairness	Intelligent systems learn from available data and they can be biased No distinction between individuals must be assured	No distinction between individuals must be applied. This could seem obvious, since it is clearly stated in the Universal Declaration of Human Rights, but it is also well known that systems based on artificial intelligence can be biased by the data used to train their models. It has already happened, in different domains, that “intelligent systems” have discriminated against some ethnic groups.
Societal and environmental wellbeing	What will be the consequences of a net impact of introducing AVs?	The use of AVs must ensure an increase in public health and mobility, reduce the traffic flow, and decrease carbon emissions. However, there is uncertainty regarding the spreading in using AVs. This could cause an increase in total pollution and congestion. The introduction of AVs must be combined with infrastructure changes aimed to facilitate and optimize the AV experience.
Accountability	Who will be responsible for any mis-happenings on or off road? Who will be punished and held guilty for accidents and breaking laws? How will the insurance company handle issues related to autonomous vehicle claims?	As previously discussed, the attribution of liability and responsibility is an open issue. Who will be responsible for the actions of the autonomous vehicle in situations where the entire control is in hands of the vehicle itself? Is it the responsibility of the human operator, the car company or the algorithms ?In case of accidents, AVs cannot be responsible, since they are not moral agents. The full deployment of autonomous vehicles on the road needs well established laws and regulations governing the liability and responsibilities. Moreover, once the vehicle’s control is in the hands of an algorithm or software, what will be the criteria to define risky and safe driving? How will the insurance companies handle the claims related to accidents and road safety issues? [101]

**Table 7:** Summary of studies that apply enabling technologies to main services and application of smart mobility.

	Autonomous Vehicles	Optimization of Logistics	Mobility-as-a-Service	Traffic Flow Optimization
Blockchain	[139,140,141]	[137,138]	[125,126,140]	[131]
Geospatial technologies	[142,143]	[133,134]	[127,128]	[133,134]
Big Data	[144,145]	[134]	[127,128,129]	[134]
Clean Energy	[146]	[132]	[130]	[132]
Artificial Intelligence	[147,148,149]	[133,134]	[126]	[133,134]
IoT	[142,145]	[135,136]	[128]	[135,136]
